# Aberrant developmental titin splicing and dysregulated sarcomere length in Thymosin β4 knockout mice

**DOI:** 10.1016/j.yjmcc.2016.10.010

**Published:** 2017-01

**Authors:** Nicola Smart, Johannes Riegler, Cameron W. Turtle, Craig A. Lygate, Debra J. McAndrew, Katja Gehmlich, Karina N. Dubé, Anthony N. Price, Vivek Muthurangu, Andrew M. Taylor, Mark F. Lythgoe, Charles Redwood, Paul R. Riley

**Affiliations:** a Department of Physiology, Anatomy and Genetics, University of Oxford, Oxford, UK; b Centre for Advanced Biomedical Imaging, Department of Medicine, University College London (UCL), London, UK; c Division of Cardiovascular Medicine, Radcliffe Department of Medicine, University of Oxford, Oxford, UK; d UCL-Institute of Child Health, London, UK; e Centre for Cardiovascular Imaging, UCL Institute of Cardiovascular Science, London, UK

**Keywords:** Thymosin β4, Titin isoforms, Developmental splicing, Dysregulated sarcomere length

## Abstract

Sarcomere assembly is a highly orchestrated and dynamic process which adapts, during perinatal development, to accommodate growth of the heart. Sarcomeric components, including titin, undergo an isoform transition to adjust ventricular filling. Many sarcomeric genes have been implicated in congenital cardiomyopathies, such that understanding developmental sarcomere transitions will inform the aetiology and treatment. We sought to determine whether Thymosin β4 (Tβ4), a peptide that regulates the availability of actin monomers for polymerization in non-muscle cells, plays a role in sarcomere assembly during cardiac morphogenesis and influences adult cardiac function. In Tβ4 null mice, immunofluorescence-based sarcomere analyses revealed shortened thin filament, sarcomere and titin spring length in cardiomyocytes, associated with precocious up-regulation of the short titin isoforms during the postnatal splicing transition. By magnetic resonance imaging, this manifested as diminished stroke volume and limited contractile reserve in adult mice. Extrapolating to an in vitro cardiomyocyte model, the altered postnatal splicing was corrected with addition of synthetic Tβ4, whereby normal sarcomere length was restored. Our data suggest that Tβ4 is required for setting correct sarcomere length and for appropriate splicing of titin, not only in the heart but also in skeletal muscle. Distinguishing between thin filament extension and titin splicing as the primary defect is challenging, as these events are intimately linked. The regulation of titin splicing is a previously unrecognised role of Tβ4 and gives preliminary insight into a mechanism by which titin isoforms may be manipulated to correct cardiac dysfunction.

## Highlights

• Thymosin β4 (Tβ4) regulates sarcomere length in the postnatal heart.
• Τβ4 KO mice have shorter thin filaments and express shorter titin isoforms.
• Τβ4 KO mice possess limited contractile reserve, in response to dobutamine stress.
• Splicing may be a novel role for Tβ4 and a target to correct cardiac dysfunction.

## 1 Introduction

The contractile properties of cardiac muscle influence the filling behaviour of the heart, determining the maximal diastolic volume, thereby providing a set point for systolic performance via the Frank-Starling mechanism [1]. Contractile function is ultimately dictated by the properties of the muscle's constituent sarcomeres, repeated units of uniform thin and thick filaments, the relaxed length of which is thought, in vertebrates, to be governed by the giant molecular spring titin [2], [3]. Sarcomere assembly is a highly orchestrated process, involving multiple protein components, which dynamically adapts throughout perinatal heart development to accommodate physiological hypertrophic cardiac growth. During this period, various myocardial proteins, including myosin heavy chain (MyHC) [4], troponins [5], tropomyosin [6] and titin [7], undergo an isoform switch from foetal to adult type in order to adjust ventricular filling. Given the association of these genes with human cardiomyopathies [8], [9], [10], [11], [12], novel genetic animal models are required to provide an understanding of the developmental sarcomere transitions that are underpinned by splicing [13].

Sarcomere assembly initiates with actin polymerization, prior to myosin incorporation [14]. Titin acts as a template to ensure the regular interdigitation and centring of thick and thin filaments; it undergoes significant isoform transition within the first few weeks of postnatal life, from the longer, more compliant foetal/N2BA isoforms to a predominance of the shorter, stiffer N2B isoform [15]. This coincides with the period of thin filament elongation to set adult sarcomere length according to the extensibility of the predominant titin isoform [2], [15]. Formation and elongation of thin filaments depends on a high intracellular concentration of monomeric actin, which is maintained in complex with actin-sequestering proteins [16]. Thymosin β4 (Tβ4) is a 43 amino acid G-actin–binding protein which functions to regulate the cellular availability of actin monomers for the formation of polymeric F-actin [17]; along with profilin, it has been shown to mediate the formation of cytoskeletal actin filaments in non-muscle cells [18]. The role of Tβ4 in striated muscle sarcomere assembly has not been examined to date, although a previous study presumed Tβ4 to be dispensable for cardiac development and function [19]. In contrast, after close examination of muscle ultrastructure and detailed cardiac phenotyping, we find that Tβ4 is required for the appropriate regulation of sarcomere length. In Tβ4 knockout mice, shortened thin filament length, associated with precocious up-regulation of the shorter isoforms of titin during the postnatal splicing transition, contribute to reduced stroke volume and reduced contractile reserve in adults. A direct role for Tβ4 was confirmed by extrapolation to in vitro culture of embryonic cardiomyocytes, whereby the titin splice defect was corrected with addition of synthetic Tβ4 to restore normal sarcomere length. The developmentally regulated titin isoform transition is frequently recapitulated, or reversed, in disease [20]; examples include elevated N2B:N2BA underlying abnormal relaxation in diastolic heart failure [21] and an up-regulation of the more compliant N2BA isoform to correct for myocardial stiffening in patients with end-stage systolic failure [22] and severe coronary artery disease [23]). The RNA binding protein RBM20, linked to human dilated cardiomyopathy [24], was found to mediate splicing of some titin exons [25]; mutations in *RBM20* resulted in persistence of the large embryonic isoform N2BA-G into adulthood. However, whether RBM20, or other splicing factors, specify inclusion of titin's N2B and N2A exons during the postnatal transition, and in disease, remains to be determined. Understanding the mechanisms that control titin splicing and identifying splicing regulators may inform approaches for therapeutic targeting and correction of cardiac dysfunction.

## 2 Materials and methods

Detailed methods are provided in the online Supplement. All procedures involving the use and care of animals were performed in accordance with the legislation of the Home Office (UK) and approved by UCL and University of Oxford Animal Welfare and Ethical Review Boards.

### 2.1 Global Tβ4 KO mice

Global Tβ4 KO mice were generated as previously described [26].

### 2.2 MR image acquisition

Mice were anaesthetised with isoflurane and maintained at 37 ± 1 °C during image acquisition. For Dobutamine (40 μg kg^− 1^ min^− 1^) and Esmolol (5 mg kg^− 1^ min^− 1^) stress tests an intraperitoneal (i.p.) infusion line was prepared and connected to an infusion pump (PHP2000 Harvard Instruments, UK). Image acquisition was performed with a 39 mm diameter volume coil (Rapid Biomedical GmbH, Germany). Cardio-respiratory monitoring and gating were performed using an MR-compatible system (SA Instruments, NY). Imaging was performed using a 9.4 T VNMRS horizontal bore scanner (Agilent Technologies, CA) with a shielded gradient system (1000 mT/m). For detailed image acquisition and analysis protocols, please refer to [27] and the online Supplement.

### 2.3 Echocardiographic and haemodynamic assessment of function

Echocardiograms were obtained in 17–18 week old male +/Y and −/Y mice under isoflurane anaesthesia. Using a Visualsonics Vevo 2100 using a 22–55 MHz transducer, pulse wave Doppler measurements were obtained across the mitral valve via the apical 4-chamber view and tissue Doppler imaging for radial velocities via a parasternal short-axis view. Haemodynamic measurements were performed on the same mice 3–4 days later under identical anaesthetic conditions. The LV was cannulated via the right carotid artery using a 1.4-F Millar Mikro-Tip cannula (SPR-839, Millar Instruments, Houston, Texas). The right jugular vein was cannulated with stretched polyethylene tubing for infusion of dobutamine hydrochloride (16 μg kg^− 1^ min^− 1^) to test contractile reserve. Measurements were obtained after 15 min of equilibration via a Powerlab 4SP data acquisition system (ADInstruments, UK). All data acquisition and analysis was performed blind to genotype.

### 2.4 Force-Ca^2 +^ sensitivity measurement

Solution compositions for force-Ca^2 +^ sensitivity measurements were determined using an iterative computer programme that uses published affinity constants [28] to calculate the equilibrium concentration of ligands and ions. Solutions contain (in mM^ 1^): 80 MOPS, 15 EGTA, 1 Mg^2 +^, 5 MgATP, 135 (Na^+^ + K^+^), 15 creatine phosphate and 20 units ml^− 1^ creatine phosphokinase, pH 7.0 (at 15 °C). Ca^2 +^ concentration was varied using CaCl_2_. Total ionic strength was 170 mM. Mice were killed via cervical dislocation. Hearts were rapidly excised and dissected in oxygenated physiological salt solution. Left ventricular trabeculae (width: 80–250 μm, length: 1.0–3.0 mm) were cut and washed overnight at 4 °C in relaxing solution containing 1% Triton X-100. After skinning, fibres were stored in 50% glycerol relaxing solution at − 20 °C for experiments within 1 week. Skinned trabeculae were assessed using an Aurora Scientific Permeabilized Fibre Test System. Sarcomere length was set at 2.0 μm in relaxing solution using laser diffraction and experiments were conducted at 15 °C. Isometric force was measured by transiently shortening the fibre to slack length. Passive force, measured in relaxing solution, was subtracted from total force at each Ca^2 +^ concentration to determine active force.

### 2.5 Adult cardiomyocyte isolation, functional assessment and sarcomere length determination

Murine left ventricular myocytes were enzymatically isolated by retrograde perfusion of the heart followed by mechanical agitation. Functional assessment was conducted, within 6 h of isolation, using an IonOptix-μStep Myocyte Contractility System. Fractional shortening was assessed via Fast Fourier Transform-based sarcomere length tracking. [Ca^2 +^] transients were measured in cells loaded with 1 mM Fura-2-AM (Life Technologies) and 2 μM Pluronic. Imaging of fixed cardiomyocytes was performed using a Leica TCS SP5 II system. Leica Application Suite (LAS) AF Lite was used to measure image intensity over a line segment drawn perpendicular to visible striations. Matlab was used to locate peaks in the intensity plot and calculate the associated difference between peaks. The mean difference between peaks for each cell was considered its sarcomere length.

### 2.6 Embryonic cardiomyocyte culture

Primary cultures of cardiomyocytes were prepared from WT or Tβ4 KO E18.5 mouse hearts, essentially as previously described for neonatal cardiomyocytes [29], and cultured at an initial density of 1 × 10^6^ cells per well in a 6-well plate (with gelatin-coated coverslips for immunofluorescence analysis). After overnight culture in plating medium, cells were washed twice in PBS and medium replaced with maintenance medium containing either PBS (vehicle control) or Tβ4 (100 ng/ml, a kind gift from RegeneRx Pharmaceuticals). Medium, supplemented with fresh Tβ4 or PBS, was changed daily and cells were harvested after 1, 5 and 9 days for real time qPCR and immunofluorescence analysis.

### 2.7 Immunodetection and histological methods

Immunofluorescence was performed on cryosections of diastole-fixed hearts or on soleus or tibialis anterior muscle and on embryonic cardiomyocytes using standard protocols and the following antibodies: sarcomeric α-actinin (SαA; Sigma), Thymosin β4 (Immundiagnostik), myomesin and cardiac myosin binding protein C (cMyBPC a kind gift of Elisabeth Ehler/Mathias Gautel), cTnT and cTNI, (Abcam); N2B, PEVK, N2A, Tmod1 and Nebulin (kind gifts of Siegfried Labeit). Images were acquired using an Olympus IX81 confocal microscope, a Zeiss AxioImager with ApoTome or a Leica DM6000 fluorescence microscope with Structured Illumination. Adult heart sections were stained with hematoxylin and eosin, using a standard protocol, or Alexa 594-conjugated wheatgerm agglutinin (Invitrogen), according to the manufacturer's instructions. Nuclear counts, cell area and cell counts were performed on sections which had been anonymised and blinded to genotype, using ImageJ software particle analysis.

### 2.8 Thin filament and sarcomere length measurements on sectioned left ventricle

Hearts were fixed in diastole by injection of 100 mM KCl into the left atrium of mice, while anaesthetised with isoflurane. After rapid arrest in diastole, 4% PFA in phosphate buffered saline (PBS) was perfused for 2–3 min before hearts were removed and incubated in the same fixative for a further 2 h. After washing with PBS and embedding in Tissue-Tek O.C.T. compound, hearts were rapidly chilled in isopentane, within a dry ice bath. 7 μm thick sections were prepared and stained, as described above. Confocal images were obtained using an Olympus IX81 confocal microscope (6 left ventricular fields of view per section from 3 sections at different levels through the heart, from 5 −/Y and 3 +/Y hearts). Images were analysed in ImageJ (http://rsb.info.nih.gov/ij), with 1D plot profiles drawn along the myofibril, perpendicular to the Z-lines. Well-defined peaks were obtained, as shown in Fig. 2T, in which major peaks represented α-actinin positive Z-lines and minor peaks represented Tmod-1 positive thin filament ends. Sarcomere length (SL) was measured as the distance between 2 α-actinin peaks (at their centres, via a perpendicular line) and TFL as the distance between an α-actinin peak and a Tmod1 peak.

### 2.9 Analysis of titin isoforms by SDS-PAGE

Hearts were homogenized in sample buffer containing 8 M urea, 2 M thiourea, 3% SDS, 75 mM DTT, 10% glycerol, bromophenol blue and 0.05 M Tris·HCl, pH 6.8 (samples prepared as described in [23]). Samples were incubated for 5 min on ice and boiled for 5 min at 95 °C, followed by centrifugation. Agarose-strengthened SDS-PAGE (2% polyacrylamide; 0.5% agarose) was performed, at 2 mA overnight, and protein bands were visualized, following Coomassie brilliant blue staining, and scanned using a ChemiDoc system (BioRad). Densitometry analysis was performed using ImageJ to determine relative intensities and % total titin.

### 2.10 RNA isolation and qRT-PCR

Total RNA was isolated from the ventricles using Trizol reagent (Invitrogen) or from embryonic murine cardiomyocytes using the RNeasy Plus Micro kit (Qiagen), and reverse-transcribed using Superscript III RT (Invitrogen). Real-time RT-PCR analysis was performed on an ABI 7900 Sequence Detector using SYBR Green (Applied Biosystems). Data were normalised against *Hprt1* expression and fold-changes determined by the 2^− ΔΔCT^ method [30]. Primer sequences can be found in the online Supplement.

### 2.11 Statistical analysis

For the comparison of LV baseline data, a two-tailed unpaired Student's *t*-test was used to determine any significant differences ( p < 0.05). The requirements for a *t*-test were assessed using a Shapiro-Wilk test for normality and an F-Test to compare the variances. For the comparison of functional indices at rest and under Esmolol treatment, a two-way ANOVA with Bonferroni correction was used. Dobutamine MRI, ECHO and haemodynamic data were analysed using a two-tailed Mann–Whitney test with multiple correction. TFL vs SL correlation was analysed by linear regression. Statistical analysis was performed using R software version 2.8.1, GraphPad Prism 5.01 or SPSS 22.

## 3 Results

### 3.1 Sarcomere length is dysregulated in Thymosin β4 KO mice

**Fig. 1:**
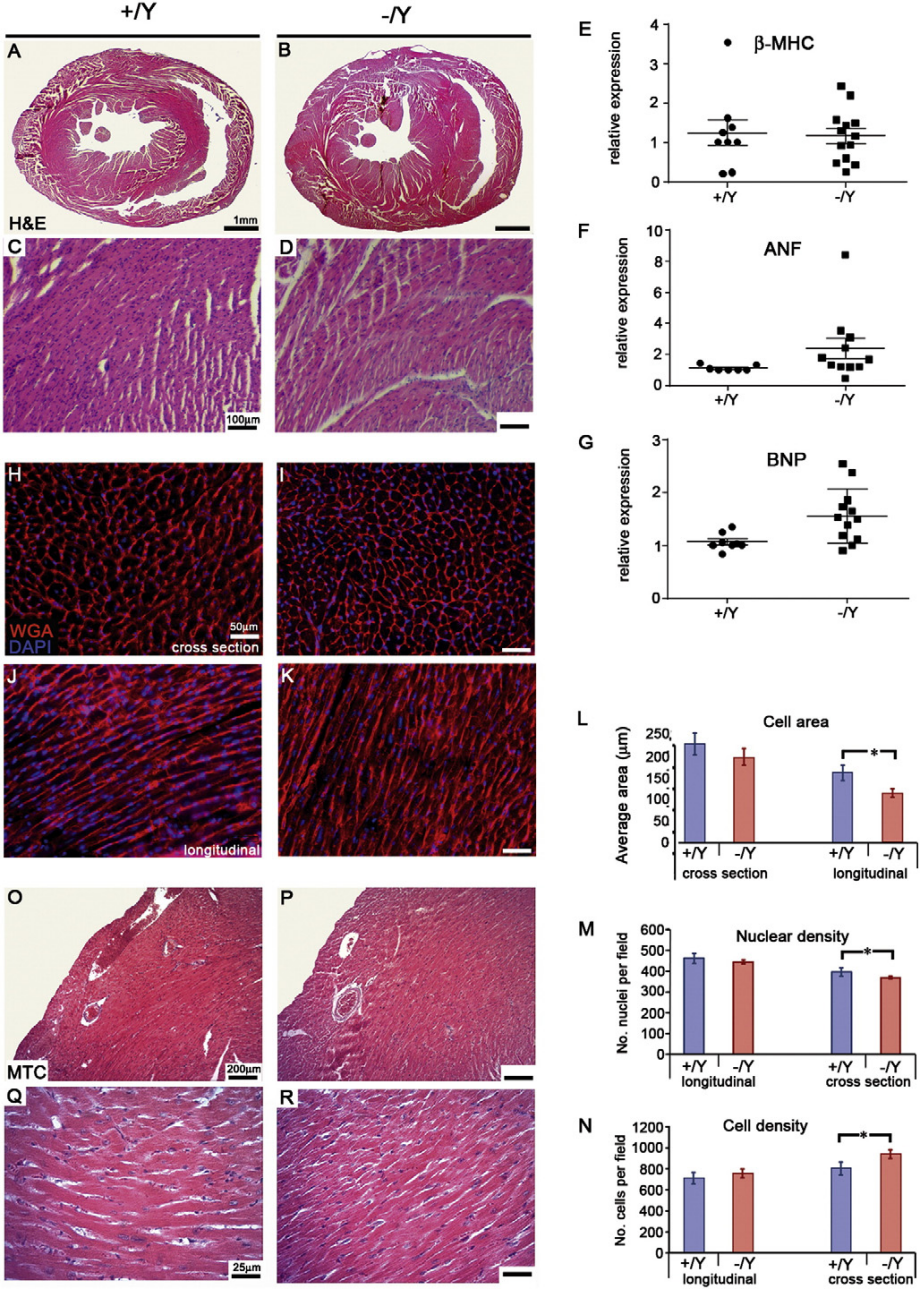
Tβ4 −/Y hearts display no hypertrophy or fibrosis although cardiomyocytes are slightly smaller. Histological sections of Tβ4 +/Y (A and C) and −/Y (B and D) littermate adult hearts reveal no evidence of hypertrophy; consistent with no significant re-expression of hypertrophy-related foetal genes, β-MHC (*Myh7*), ANF (*Nppa*) or BNP (*Nppb*) (E–G). Quantitative analysis of Tβ4 −/Y myocardium in transverse section revealed a significant reduction of myocyte cross-sectional (I, compared with +/Y, H) and longitudinal area (K, compared with J), with more cells at a reduced size compared with +/Y controls (quantification in L–N). Masson's Trichrome staining revealed no evidence of interstitial or perivascular fibrosis (O–R). Scale bars: 1 mm in A applies also to B; 100 μm in C applies to D; 50 μm in H applies to I, J, K; 200 μm in O applies to P and 25 μm in Q applies to R; n = 6 hearts per genotype, except E–G: n = 9 +/Y; n = 13 −/Y.

Global loss of Tmsb4x in a mouse model causes a partially penetrant hemorrhagic vascular phenotype and loss of 20% of hemizygous null males (−/Y) prior to birth, due to mural cell insufficiency [26]. We sought to examine the consequences of Tβ4 loss on myocardial histology and function in surviving −/Y adult mice. At the level of gross anatomy and hematoxylin/eosin staining of diastole-fixed transverse sections, hearts displayed no overt abnormality (Fig. 1). Histologically there was no explicit indication of hypertrophy (Fig. 1, A–D), although chamber volumes appeared marginally smaller in −/Y mice. Accordingly, no differences were recorded in MRI-measured left ventricular mass (Table A1), nor was there any significant re-expression of hypertrophy-related foetal genes, β-MyHC (*Myh7*), ANF (*Nppa*) or BNP (*Nppb*) (Fig. 1, E–G). A modest, yet significant, reduction of myocyte cross-sectional area was detected in Tβ4 −/Y hearts in transverse section, with more cells at a reduced size per imaged field, compared with +/Y controls (Fig. 1, H—N). Masson's Trichrome staining revealed no evidence of interstitial or perivascular fibrosis (n = 6 hearts per genotype; Fig. 1, O–R).

**Fig. 2:**
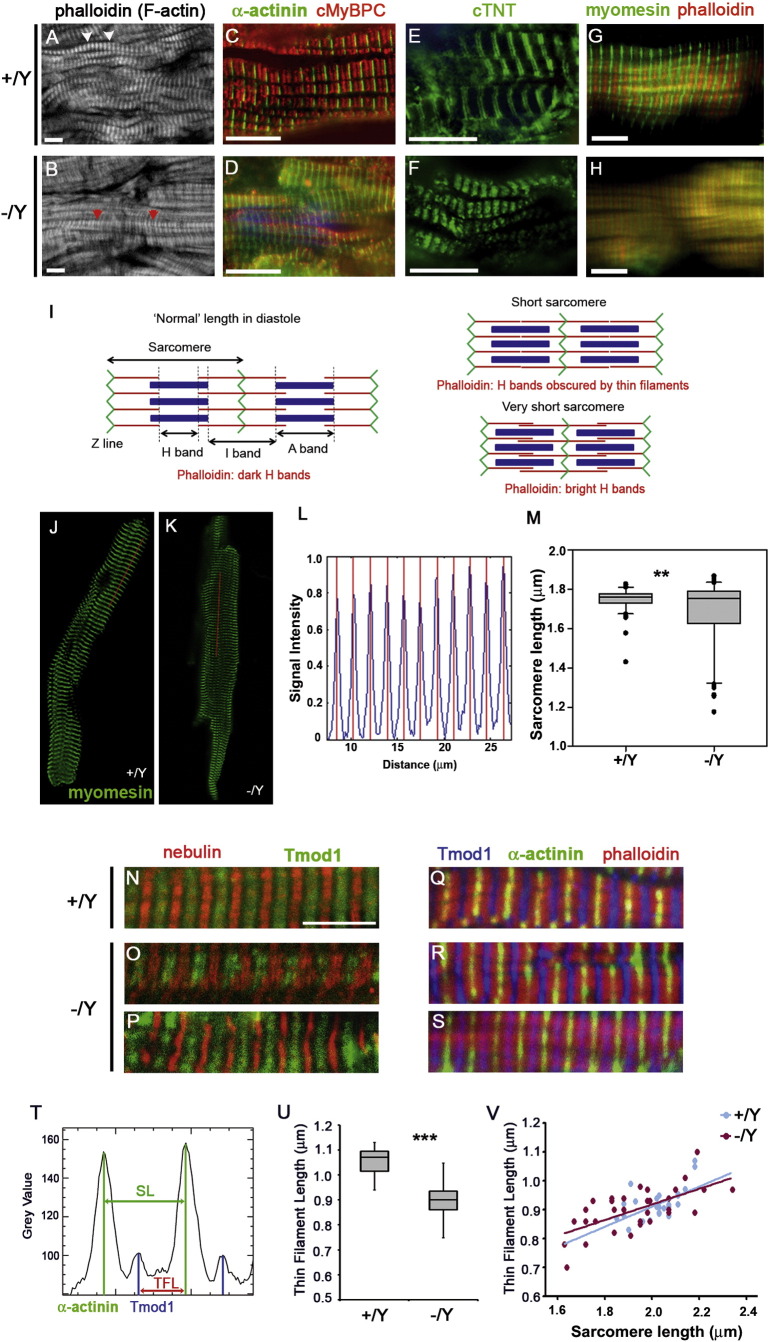
Dysregulated sarcomere length in Tβ4 −/Y myocardium. Assessment of sarcomeric components in diastole-fixed adult heart sections (n = 5 per genotype): filamentous actin (A, B), α-actinin/cMyBPC (C, D), cardiac troponin T (cTNT; E, F), myomesin/phalloidin (G, H). Staining of polymerized actin with phalloidin revealed that > 60% of −/Y cardiomyocytes contained shortened sarcomeres (B), compared with +/Y (A). Schematic in I illustrates how short sarcomeres in −/Y hearts present with additional phalloidin bright bands (red arrowheads in B) compared with normal diastolic sarcomeres in +/Y hearts (white arrowheads in A), in which clear dark bands are seen either side of the Z line. Shorter sarcomere length was also evident upon visualisation of Z-disc, thick filament proteins (α-actinin, D, compared with C; cMyBPC, D, compared with C; myomesin, H, compared with G), and components of the troponin complex (cTNT, F, compared with E) and M band (H, compared with G). Representative images of cardiomyocytes from +/Y (J, n = 55) and −/Y (K, n = 63) mice. Sarcomere length was assessed by finding peaks within a plot of image intensity (L) over a line perpendicular to striations (J, K) and revealed a negative skew in its distribution. Mean sarcomere length was determined for each cell and the population of means illustrated via box-and-whisker (M). Means (1.75 μm +/Y, 1.68 μm −/Y) were significantly different by 1-way ANOVA (p < 0.01). Thin filament length (TFL) was assessed by examining nebulin/Tmod1 banding (N—P) and measured as distance between the peaks, in intensity profile, of Tmod1 and α-actinin (Q–T). In keeping with shortened sarcomere length in −/Y hearts, TFL was found to be more variable and, overall, significantly shorter in −/Y hearts (U; 0.98 ± 0.09 μm vs. 1.06 ± 0.05 μm in +/Y hearts; p < 0.001 by 1-way ANOVA). TFL correlates with SL in both +/Y and −/Y hearts (V). By linear regression analysis, both slopes were significantly different from zero (+/Y: 0.0006 and −/Y: < 0.0001) but were not significantly different between genotypes, in terms of slope, elevation or intercept. Scale bars: A–H: 10 μm; N (applies to N–S: 5 μm). (For interpretation of the references to colour in this figure legend, the reader is referred to the web version of this article.)

Due to the role of Tβ4 in regulating the availability of G-actin, required for thin filament formation, we focused our analyses on the cardiac sarcomere. Assessment of the major components was performed using immunofluorescence on transverse diastole-fixed adult heart sections. Phalloidin stains thin filaments of the I band which extend as bands of reduced brightness either side of the Z line (bright banding, white arrowheads, Fig. 2A; schematic Fig. 2I). H bands, which contain only thick filaments, appear as distinct dark bands between phalloidin-stained I bands and were clearly discerned in most +/Y cardiomyocytes. In contrast, sarcomeres in −/Y hearts appeared either marginally shorter, in which dark H bands were not detectable, or considerably shorter, which manifested as additional phalloidin bright bands in between Z lines, due to thin filament overlap (red arrowheads, Fig. 2B; schematic Fig. 2I). Shorter sarcomere length was also evident in −/Y cardiomyocytes upon visualisation of other sarcomeric proteins, including thick filament components, such as cardiac myosin binding protein C (cMyBP-C, Fig. 2C, D), as well as the Z-disc protein α-actinin (Fig. 2C, D), constituents of the troponin complex (cTNT, Fig. 2E, F) and the M band protein myomesin (Fig. 2G, H). Further evidence of shorter sarcomeres was obtained from double staining for components of the thin and thick filaments. When stained with α-actinin and cMyBPC, +/Y myofibrils demonstrated regular, discrete alternating Z lines and A bands (Fig. 2C). Similarly organised were the alternating M line - thin filament markers, myomesin and phalloidin (Fig. 2G). In contrast, −/Y myofibrils displayed abnormally close apposition of Z lines-A bands (α-actinin/cMyBPC, Fig. 2D) and M line – thin filaments (myomesin/phalloidin, Fig. 2H), in > 60% of cardiomyocytes examined (quantification based on image analysis of n = 6 hearts per genotype; 8 left ventricular fields of view per section from 3 sections at different levels through the heart). The shorter sarcomere length in −/Y myofibrils was statistically significant when quantified (1.81 ± 0.09 in +/Y myofibrils vs 1.52 ± 0.21 in −/Y; p < 0.001, based on phalloidin staining; Z disc to Z disc measurements on 10 cardiomyocytes per field, from 8 left ventricular fields of view per section; 3 sections at different levels through each heart; n = 6 hearts per genotype). Greater variance in sarcomere length was observed in −/Y cardiomyocytes; notably, myocytes with shorter sarcomeres were not uniformly distributed throughout the left ventricle, rather the 62.4% of affected myocytes clustered in patches.

In myocardial sections, the angle of tissue relative to the imaging plane may confound accurate measurement of sarcomere length. We therefore isolated and imaged fixed cardiomyocytes stained with an anti-myomesin antibody (Fig. 2J and K) and quantified sarcomere length as the distance between peaks across a line perpendicular to striations (Fig. 2J–L). The mean sarcomere length was significantly shorter in −/Y cardiomyocytes (1.75 μm +/Y, n = 55; 1.68 μm −/Y, n = 63; p < 0.01) and a striking degree of variability in −/Y sarcomere length was observed (inter-quartile range 1.64–1.79 μm in −/Y versus 1.73–1.78 μm in +/Y; Fig. 2M), confirming our measurements in sectioned hearts, with some as short as 1.18 μm.

On the basis of these findings and the established role for Tβ4 in regulating actin filament formation, we sought to directly determine whether thin filament length (TFL) was altered in Tβ4 −/Y hearts, using immunofluorescence based assays with quantitative image analysis. Firstly, we stained thin filaments using an antibody against an extreme N-terminal epitope of nebulin, a large protein that spans the length of the thin filament and which was originally proposed to act as a “thin filament ruler” by determining where tropomodulin (Tmod) caps the slow-growing, pointed end [31]. Along with dysregulated sarcomere length in −/Y hearts, we found TFL to be correspondingly affected. Whereas uniform nebulin banding was observed in +/Y hearts (Fig. 2N), variation was observed in −/Y hearts, with ~ 10% of cardiomyocytes demonstrating thicker banding (Fig. 2O) and ~ 25% demonstrating thinner banding (Fig. 2P). Although suggestive of a shortened thin filament phenotype, recent studies have questioned the validity of nebulin as a marker for thin filament ends [32], [33], [34], [35]. For accurate thin filament measurements and quantification, we directly determined TFL based on the distance between the pointed end (Tmod1) and the Z disc (α-actinin; Fig. 2Q–U), as used previously [36], [37]. Quantification of distance between peaks in intensity plots (Fig. 2T) was performed using Image J (n = 3 +/Y; n = 5 −/Y hearts per genotype; 6 left ventricular fields of view per section from 3 sections at different levels through the heart). By this method, +/Y TFL measured 1.06 ± 0.05 μm (Fig. 2Q, T, U) whereas −/Y TFL was 0.98 ± 0.09 μm, revealing a more variable, and generally shorter, TFL (Fig. 2R–U). Examples of moderately short and extremely short TFL are shown in Fig. 2R and Fig. 2S, respectively. By measuring sarcomere length (SL), as the distance between α-actinin-labelled Z lines, on the same myofibrils, we found that TFL correlates with SL in both +/Y and −/Y hearts (Fig. 2V). Equations were TFL = 0.35 SL ± 0.21 and TFL = 0.27 SL ± 0.38, for +/Y and −/Y, respectively. Greater range and variance in both TFL and SL were seen in −/Y hearts, compared with +/Y hearts. However, by linear regression analysis, there were no significant differences between genotypes, in terms of slope, elevation or intercept, and both slopes were significantly different from zero (+/Y: 0.0006 and −/Y: < 0.0001).

**Fig. 3:**
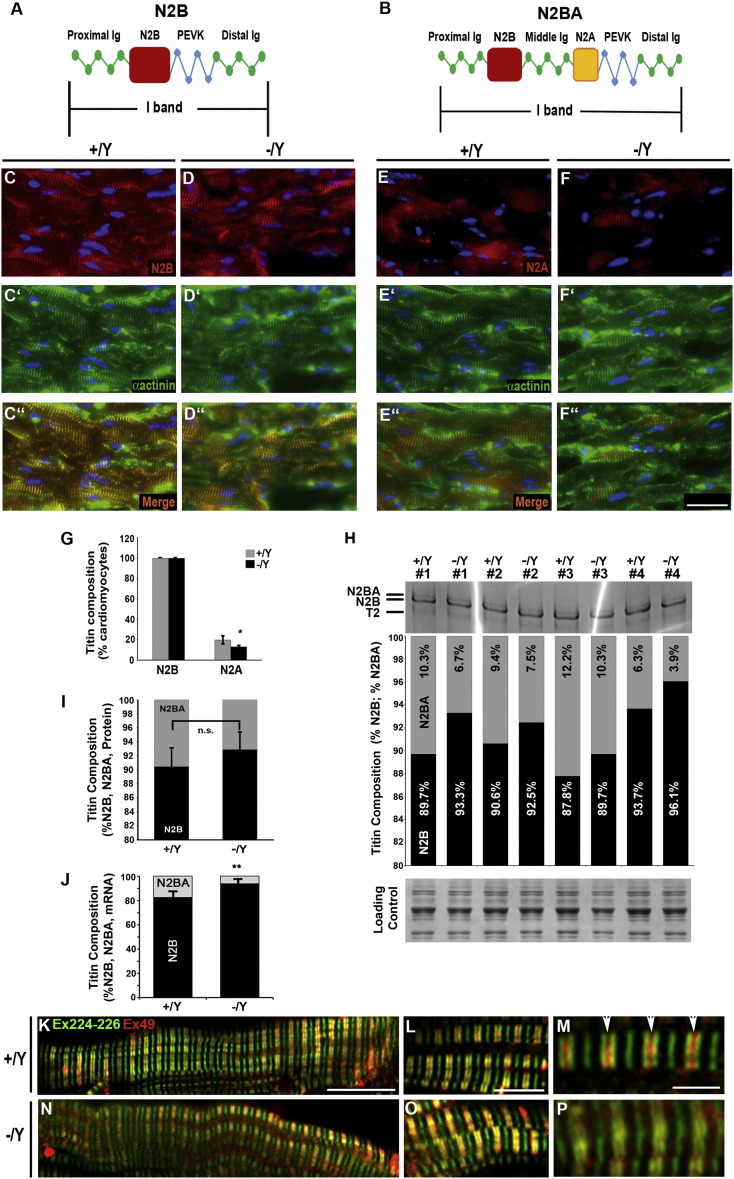
Shortened sarcomere length in Tβ4 −/Y cardiomyocytes is associated with altered expression of titin isoforms. Titin isoforms containing the N2B element (A, B) were strongly expressed in all cardiomyocytes of both +/Y and −/Y hearts (n = 5 each; C, D, co-expressed with α-actinin, C′, C″, D′, D″,G). In contrast, the N2A element (B), present only in N2BA titin, was expressed at detectable levels in only 19.8% of +/Y (E, G) and 12.9% of −/Y cardiomyocytes (F, G; p < 0.5). Separation of N2B and N2BA isoforms on agarose-strengthened SDS-2% polyacrylamide gels reveals a modestly increased %N2B, with considerable variability. Mean %N2B was not statistically different: 90.5 ± 2.5% (+/Y) vs 92.9 ± 2.6% (−/Y), (H, I; n = 4). Tβ4 −/Y ventricles express a higher ratio of N2B:N2BA titin (J, p < 0.001; n = 8), determined by qRT-PCR. Dual immunofluorescence of titin exons 49 (N2B; red) and 224–226 (PEVK segment; green) confirms a reduced length of the variable Ig domain-containing spring region in −/Y hearts (N—P), compared with +/Y hearts (K–M). White arrowheads indicate position of Z lines. 20 μm scale bar in F″ applies to C—F. 10 μm, 5 μm and 2 μm in K, L, M, respectively apply to N, O and P. Error bars in G, H: SEM. (For interpretation of the references to colour in this figure legend, the reader is referred to the web version of this article.)

Although it continues to be debated [38], [39], [40], [41], [42], [43], a number of studies support the notion that titin is the primary determinant of relaxed sarcomere length [2], [44], present during the earliest stages of myofibril assembly [14]. We therefore sought to determine the relative expression of titin isoforms in Tβ4 −/Y and control hearts. Two main titin isoform classes are expressed in mammalian myocardium [45]. Splicing of exons 49/50 to exon 219 produces the relatively short and stiff N2B isoforms while additional inclusion of exons 102 to 111 (coding for the N2A element), produces the larger and more compliant N2BA class, with its longer PEVK segment and additional extensible Ig domains (Fig. 3A and B). N2B and N2BA isoforms are co-expressed in the cardiac sarcomere; their relative expression determines myofibrillar extensibility and contractility of the myocardial walls. We investigated isoform expression at the protein level using antibodies against the N2A and N2B elements [46]. Since the N2B element exists in both classes of isoforms, N2B-positive sarcomeres were readily detected in all cardiomyocytes (Fig. 3C and D), in keeping with the reported expression in rodent hearts [47]. In contrast, N2BA titin (detected with anti-N2A) was only weakly expressed and at detectable levels in only 19.8% and 12.9% of +/Y and −/Y cardiomyocytes, respectively (p < 0.05, n = 5, Fig. 3E–G). In an attempt to quantify the ratio of N2B:N2BA isoform expression, protein from +/Y and −/Y hearts were resolved on an agarose-strengthened polyacrylamide gel and stained with Coomassie brilliant blue (Fig. 3H). Gels were digitized and densitometry analyses were performed using ImageJ (Fig. 3H, I). Although variable, % N2B of total titin was typically higher in −/Y hearts, in the range of 89.7%–96.1%, compared with 87.8%–93.7% in +/Y hearts. The means 90.5 ± 2.5% (+/Y) vs 92.9 ± 2.6% were not statistically different, likely because of variability and low sensitivity of this technique (as N2B titin represents 90–95% total titin in adult rodent hearts, there is little scope to detect weak N2BA bands (~ 5%) without saturating the stronger N2B band). To overcome these difficulties, we devised a qRT-PCR method to quantify the expression levels of the alternatively spliced N2A and N2B elements from total myocardial RNA (based on a similar qRT-PCR assay for rat N2B/N2BA isoforms [48]). In keeping with shorter sarcomere length, and consistent with the isoform-specific antibody staining, Tβ4 −/Y hearts expressed a significantly higher proportion of the short N2B isoform mRNA (Fig. 3J; 93.7 ± 1.3% N2B vs 82.4 ± 3.1% N2B in + Y; p < 0.001; n = 8). More direct evidence of shortened titin in −/Y hearts was sought using antibodies against exons 49 (N2B) and 224–226 (PEVK segment) to assess the length of the variable Ig domain-containing region. As shown in Fig. 3 K–M, discrete Ex224(green)-Ex49(red)-Ex49(red)-Ex224 (green) banding can be observed around each Z line (white arrowheads) in +/Y hearts, whereas the banding cannot be resolved to the same extent in −/Y hearts (Fig. 3N–P, confirming a shorter spring region in −/Y titin, characteristic of the shorter N2B isoform. Cardiomyocytes that express high levels of N2B titin, relative to N2BA titin, have a higher passive stiffness, which limits their maximal slack length [45].

Dysregulated (generally shorter) thin filament and titin length were the principal measurable defects in −/Y hearts. Analysis of the Ca^2 +^ regulation of force production using demembranated trabeculae isolated from wild type and knockout mice showed very similar Ca^2 +^-sensitivity (pCa_50_ 5.94 ± 0.01 and 5.92 ± 0.01 for +/Y and −/Y, respectively) and therefore the sarcomere length differences are very unlikely to be due to Ca^2 +^-sensitivity discrepancies. In isolated cardiomyocytes, we detected no noticeable differences in fractional shortening, nor in Ca^2 +^ transient magnitudes (Fig. A1).

### 3.2 Loss of Thymosin β4 impacts cardiac function

**Fig. 4:**
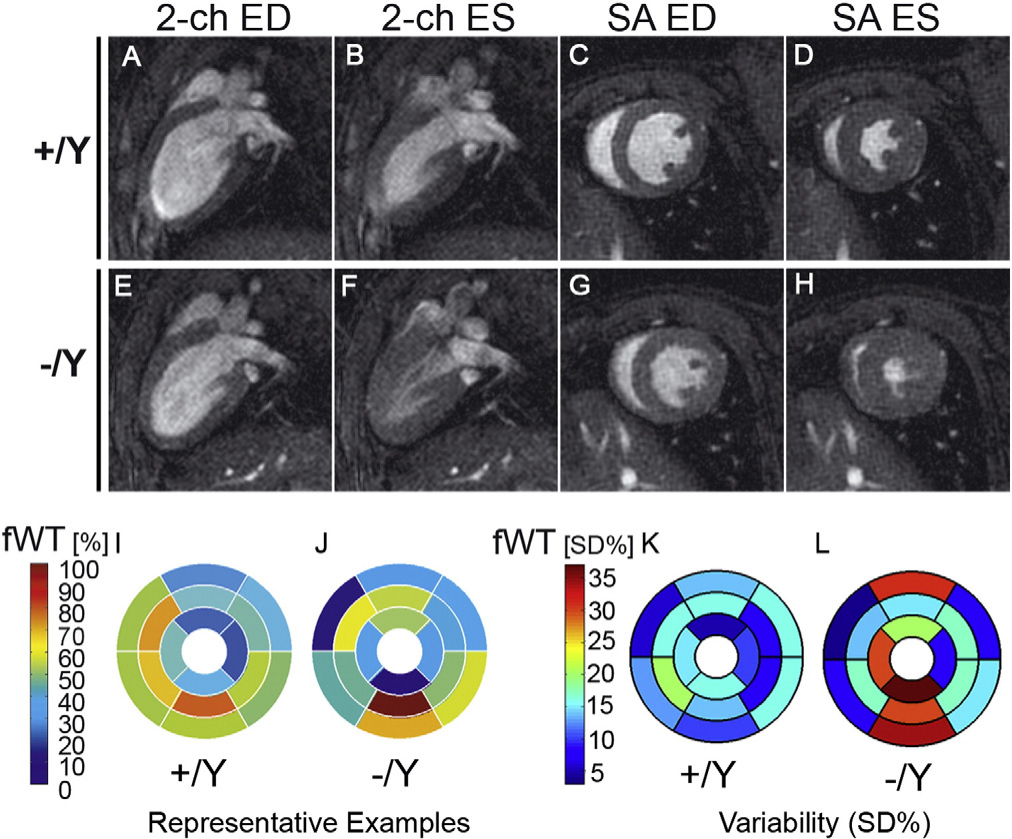
Thymosin β4 knockout hearts show reduced ventricular volumes and regional variability in wall motion. Representative two-chamber long axis (2-ch; A, B, E, F) and mid ventricular short axis (SA; C, D, G, H) images at end diastole (ED) and end systole (ES) for +/Y (A–D) and −/Y (E–H) mice. Images reveal small ED and ES volumes in −/Y mice. Bulls eye plots reveal a high local variability in fractional wall thickening (fWT) in −/Y LV myocardium (representative example in J, compared with +/Y, I). Segment-wise comparison of variances (% standard deviation (%SD) for +/Y (K) and −/Y (L) LV (Table A3; n = 8 +/Y; n = 9 −/Y), revealed greater variability in fWT in anterior (p < 0.05) and inferior segments of –Y LV (L, compared with K) while septal and lateral segments displayed reduced variability (Table A3).

Given the variable sarcomere length in Tβ4-null hearts, we sought to investigate the effect of Tβ4 loss on cardiac function. High temporal resolution cine-MRI was performed to assess global functional and morphological parameters in four month old +/Y and −/Y mice (Fig. 4; Table A1). Both left ventricular (LV) end-diastolic (EDV) and end-systolic volumes (ESV) were significantly smaller in −/Y mice (EDV: 57.4 ± 2.2 vs 67.7 ± 1.8 μl in +/Y mice; ESV: 16.9 ± 1.5 vs 23.2 ± 1.4; p < 0.001, n = 14–16), as apparent from 2-chamber long axis (Fig. 4A, B (+/Y) and E, F (−/Y)) and mid-ventricular short axis images (Fig. 4C, D (+/Y) vs G, H (−/Y)). The combined effect was a lower stroke volume in −/Y mice (SV; Video 1 online and Table A1), but with an elevated ejection fraction (EF; Table A1). Consistent with these data, a trend towards higher dP/dt_max_ upon LV haemodynamic assessment suggests higher baseline contractility in −/Y mice (Table A2). Increased heart rate was consistently observed in Tβ4 − Y mice across multiple independent experiments (Table A1, A2, A4 and A5) and, while this did not reach statistical significance in any single experiment, the effect, coupled with increased EF (p < 0.05), was sufficient to maintain a near-normal cardiac output (CO, Table A1).

When segmentation techniques were applied, greater regional variability in % fractional wall thickening (fWT) was apparent in −/Y hearts. Whereas sector values for +/Y hearts generally ranged from 25% to 75%, a significantly wider range of values (5% to 95%) were observed in −/Y mice (examples in Fig. 4I and J), with considerable variation both within and between hearts. When fWT standard deviation (SD) was compared for all +/Y and −/Y LV (Fig. 4K and L; Table A3; n = 8 +/Y; n = 9 −/Y), a segment-wise comparison of variances revealed that variability was greater in −/Y within anterior (p < 0.05) and inferior segments, while septal and lateral segments displayed less variability. When all fWT SD of +/Y vs −/Y were compared, using an F-test, a p-value of < 0.01 was obtained. Variation in wall motion contributed to a more pronounced twisting motion in −/Y hearts upon contraction (Video 1 online).

### 3.3 Tβ4 −/Y mice possess a limited contractile reserve

**Fig. 5:**
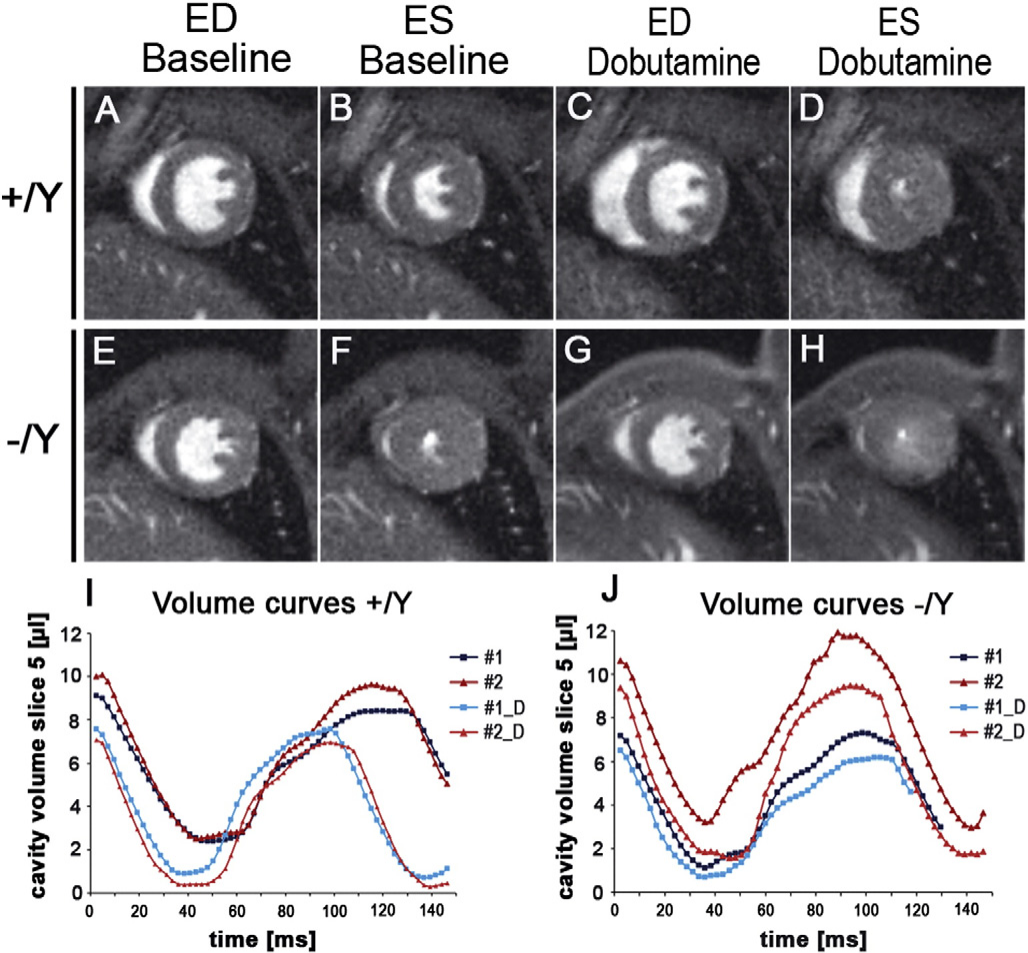
Tβ4 −/Y mice possess limited contractile reserve. Mid-ventricular short axis images, at baseline and under dobutamine stress, were acquired at high frame rate. Tβ4 +/Y: A–D; Tβ4 −/Y: E–H. End systolic volume at baseline of Tβ4 −/Y mice (F) is considerably reduced compared with control (+/Y) mice (B; Table A4; n = 4 +/Y, 6 −/Y), limiting the extent to which LV contractility can be further increased under dobutamine stress (Tβ4 +/Y: D; Tβ4 −/Y: H). Representative volume curves for +/Y (I) and −/Y mice (J) demonstrate reduced contractile reserve (characteristic leftward shift, as observed in +/Y) in −/Y mice upon β-adrenergic stimulation (shown for n = 2 each genotype).

**Fig. 6:**
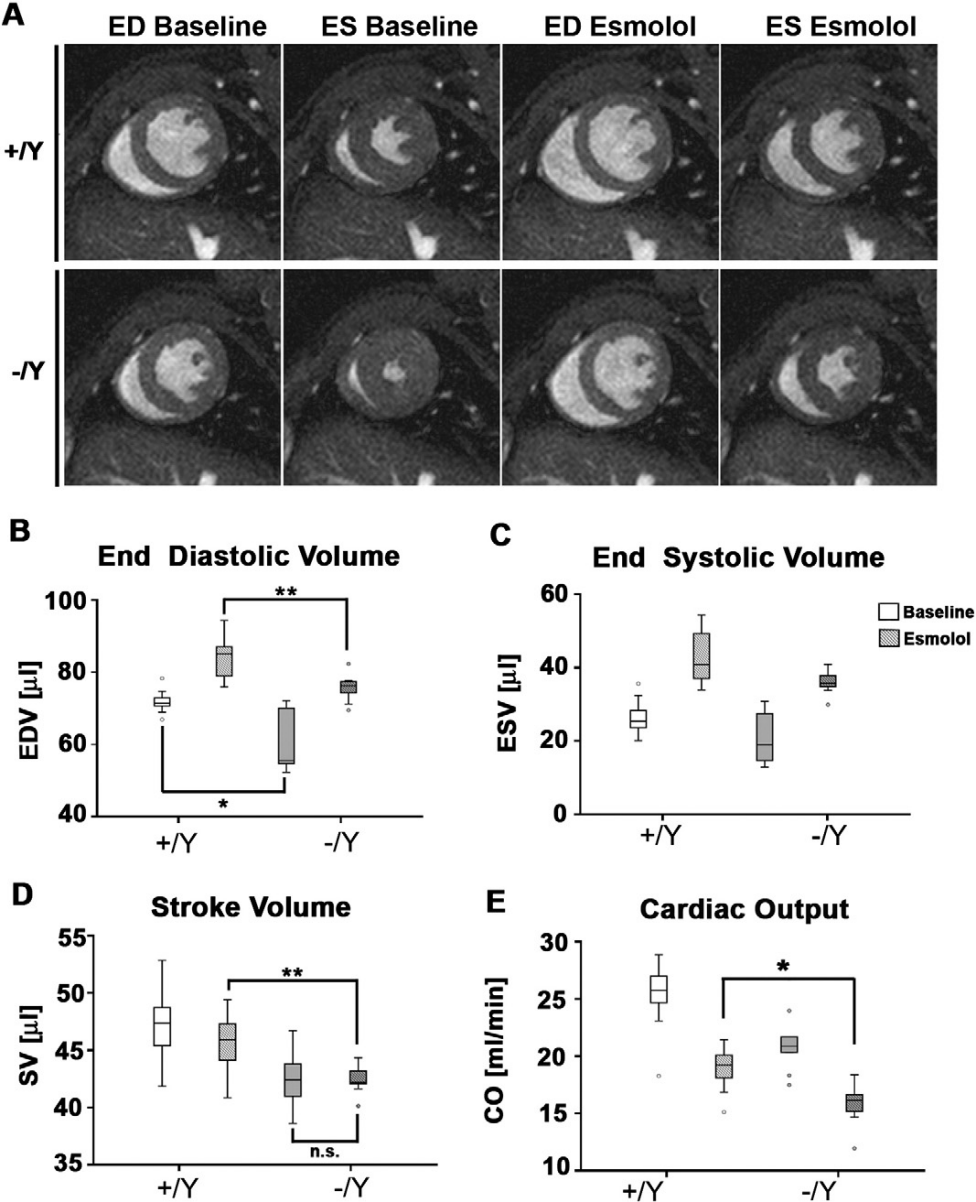
Tβ4 −/Y mice are unable to adequately alter stroke volume or maintain cardiac output during β-blockade. Representative mid ventricular short axis slices showing that Esmolol treatment increased EDV and ESV in both +/Y and −/Y hearts (A–C; Table A5). Esmolol treatment (hatched boxes) reduced stroke volume (SV) in +/Y mice, whereas −/Y mice were unaffected (D), and cardiac output dropped significantly compared with that of +/Y mice (E). *: p < 0.05; **: p < 0.01. n = 9 +/Y, 9 −/Y.

Cardiac dysfunction may only become apparent during physiological or pathological stress. To simulate the effect of exercise, we assessed cardiac performance in Tβ4 knockout mice subjected to dobutamine treatment, with high temporal cine MR imaging (n = 4 +/Y; 6 −/Y Fig. 5; Table A4; Video 2 online). The inotropic effects of dobutamine, mediated primarily by β_1_-adrenergic receptor activity, increase cardiac output via elevated heart rate and enhanced myocardial contractility. +/Y control mice (Fig. 5A–D) demonstrated a 15% increase in heart rate (+ 87 bpm; Table A4), decreased ESV and 27% increased EF (Fig. 5A–D). The characteristic leftward shift in the time/volume curve (Fig. 5I) is evidence of a normal contractile reserve in +/Y mice. In contrast, −/Y mice (Fig. 5E–H) already display small ESV (Fig. 5F), elevated heart rate and ejection fraction at baseline, with left-shifted curves, compared with +/Y (Fig. 5J). The small ESV in −/Y only modestly decreased with dobutamine treatment (compare Fig. 5F and H versus Figs. B and D); no discernible leftward shift was observed (Fig. 5J). EF was elevated by only 10% (p = 0.029 ΔBaseline +/Y vs −/Y; Table A4) and heart rate by only 5% (35 bpm; Table A4). Consistent with a trend towards reduced ΔdP/dt max by haemodynamic analysis (Table A2), these data suggest that, although maximal contractile function per se is not impaired, Tβ4 −/Y mice are restricted in their capacity to increase contractility when required; −/Y mice appear to utilise a proportion of “normal” contractile reserve at baseline, to compensate for their reduced end-diastolic volumes. Tβ4 −/Y mice depend upon increasing heart rate and EF to compensate for small EDV and maintain an effectively normal cardiac output (CO; Table A1). Moreover, their limited contractile reserve suggests they are unable to alter stroke volume. We, therefore, predicted that −/Y mice would struggle to maintain CO during β-blockade and sought to test this hypothesis by bolus infusion of Esmolol (β1-selective). Esmolol treatment slowed heart rate and increased EDV and ESV in both +/Y and −/Y hearts (n = 9 +/Y, 9 −/Y; Fig. 6A–C; Table A5; Video 3 online). The reduction in SV upon Esmolol treatment (Δ baseline), was significantly diminished in −/Y mice (Fig. 6D; Table A5; p = 0.05, Mann Whitney) and CO became significantly reduced in −/Y mice under Esmolol treatment (Fig. 6E; Table A5; p < 0.05, ANOVA).

Tβ4 −/Y mice share some features of the murine R193H cTnI mutation model of restrictive cardiomyopathy (RCM) [49]; namely, short ED sarcomere length, regionalized increases in fractional wall thickening, reduced EDV, an inability to increase cardiac output in response to β-adrenergic stimulation or to reduce stroke volume in response to β-blockade. We therefore assessed haemodynamic function by pressure-volume loop analysis (Table A2) and by Doppler echocardiography (Table A6) but found no abnormalities either in pressure, or in filling, by the respective techniques, to definitively confirm a phenotype of diastolic dysfunction or RCM in Tβ4 −/Y mice.

### 3.4 Sarcomere length is also dysregulated in skeletal muscle of Thymosin β4 KO mice

In order to determine whether the shortened sarcomere phenotype in Tβ4 KO mice is specific to the heart, we examined skeletal muscle for evidence of a similar dysregulation. Soleus and tibialis anterior (TA) muscles from Tβ4 −/Y mice (n = 5) and +/Y controls (n = 3) were sectioned in a longitudinal plane and stained for multiple sarcomere proteins. Shortened sarcomere length was evident in both soleus and TA muscles from −/Y mice (data from soleus shown in Fig. A2). Closer apposition of Z- and M- bands was revealed with dual myomesin/phalloidin staining (A +/Y vs B −/Y); shortened titin was confirmed by the reduced spacing of the N2A element (C +/Y vs D −/Y). We conclude that the requirement for Tβ4 for appropriate sarcomere/titin length is not specific to the cardiac muscle but common to postnatal striated muscle types.

### 3.5 Dysregulation of sarcomere length and titin isoform transition occur in Tβ4 KO mice during postnatal cardiac growth

**Fig. 7:**
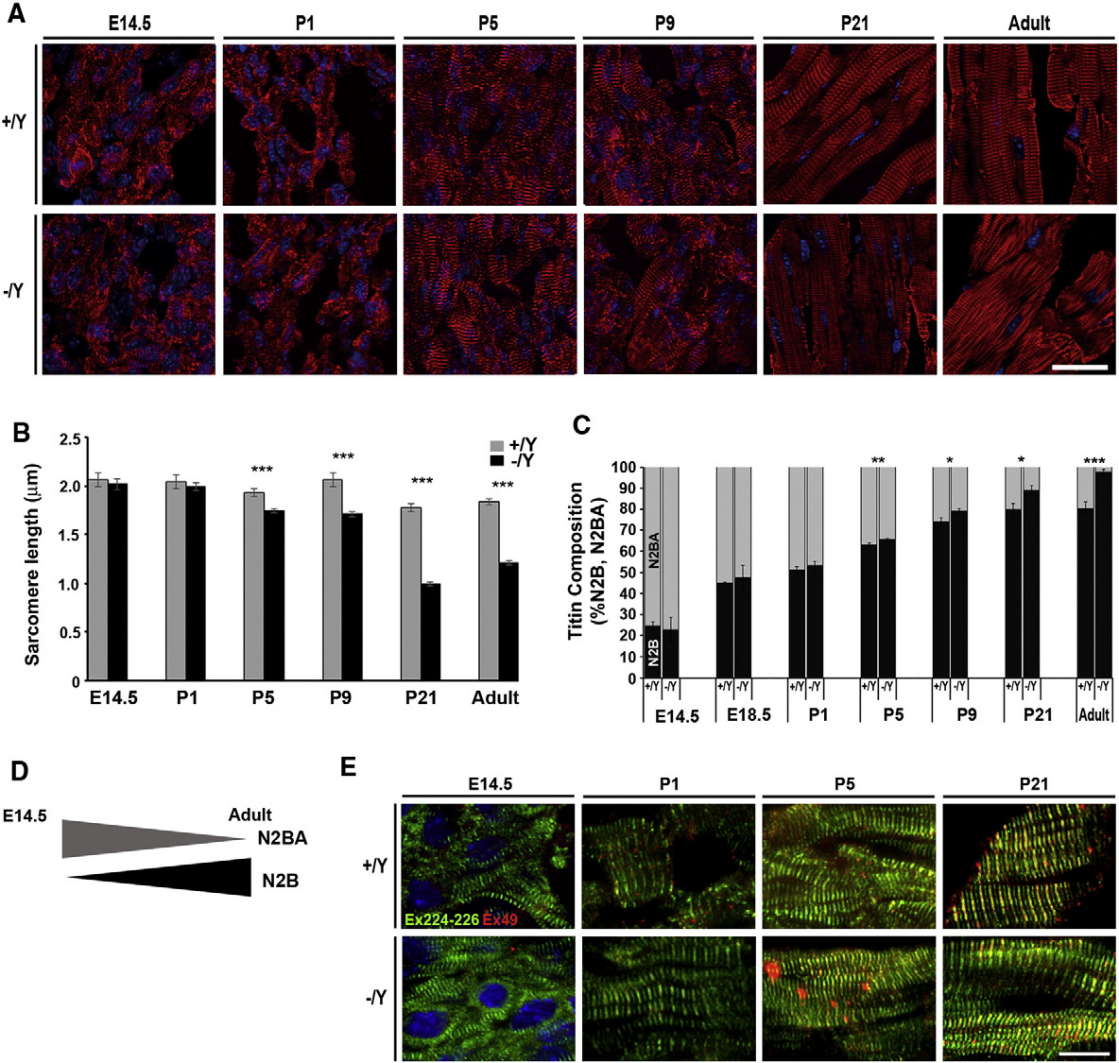
Dysregulation of sarcomere length and titin isoform transition occur in Tβ4 KO mice during postnatal cardiac growth. Tracking sarcomere maturation through late embryonic and postnatal development (A, phalloidin staining) reveals the earliest evidence of shortened sarcomeres in −/Y hearts at P5 (B; p < 0.001; n = 3–7 per genotype, per stage). +/Y embryos displayed the expected postnatal transition from predominantly N2BA titin to predominantly N2B titin (C, D). This was emulated in −/Y embryos until P1, at which point the shorter N2B isoform became precociously up-regulated and continued to be expressed at an elevated ratio in −/Y hearts throughout later development (C). The titin transition visualized by dual immunofluorescence with exon 49/exon 224 (N2B, red/PEVK segment, green) antibodies (E). Discrete N2B/PEVK banding, around the Z line, is seen in +/Y hearts throughout the P1-P5-P21 transition, whereas, in −/Y hearts, bands become closely apposed from P5 onwards, such that discrete bands could not be discerned. Scale bar in A (all panels): 20 μm. Scale bar in E (all panels): 10 μm *: p < 0.05; **: p < 0.01; ***: p < 0.001. Error bars in B, C: SEM. (For interpretation of the references to colour in this figure legend, the reader is referred to the web version of this article.)

Having demonstrated a shorter PEVK spring region and an elevated ratio of N2B:N2BA titin in −/Y adult hearts, consistent with shortened sarcomeres and altered function, we sought to track the postnatal titin isoform transition, together with sarcomere length, over the course of development to determine the earliest appearance of the phenotype. It has been shown that embryonic day 16 (E16) rat hearts exclusively express a long and compliant N2BA titin but no N2B titin [15]. Around birth, down-regulation of the N2BA isoform coincides with up-regulation of the less extensible N2B isoform to reach > 90% N2B by around P21, thereby increasing titin-based passive stiffness. In diastole-fixed sectioned hearts, −/Y sarcomeres, were indistinguishable from +/Y controls until postnatal day 5 (P5; Fig. 7A; B; n = 3–7 per genotype, per stage) p < 0.001). By P21 and into adulthood, sarcomeres were 30–40% shorter in −/Y mice (p < 0.001, Fig. 7A–B). +/Y embryos displayed the expected transition from exclusively N2BA titin to predominantly N2B titin during late embryonic and early postnatal development (Fig. 7C, D). Since N2BA titin contains both N2B and N2A elements, the qRT-PCR-based assay does not reflect the complete absence of N2B that has been demonstrated in embryonic heart but accurately tracks the N2BA-N2B transition in relative terms. An equivalent N2BA-N2B switch was observed in −/Y embryos from E14.5 through to P1 (Fig. 7C), at which point the less compliant N2B became precociously expressed, to significantly elevated levels by P5, and remained proportionally higher in −/Y hearts throughout later development (Fig. 7C, D). The titin transition was also monitored by dual immunofluorescence with exon 49/exon 224 (N2B/PEVK segment) antibodies. In +/Y and −/Y hearts, maturing sarcomeres show appropriate organisation of N2B/PEVK banding, around the Z line, by P1, although, unexpectedly, N2B staining was comparatively weaker in both mutant and controls at stages pre-P5 (Fig. 7E). Whereas the discrete banding remained visible in +/Y hearts throughout the P1-P5-P21 transition, the N2B and PEVK bands in −/Y hearts became closely apposed from P5 onwards, such that discrete bands could not be discerned. Thus, the shortened sarcomere phenotype in Tβ4 −/Y mice arises early in postnatal life, coincidentally with precocious up-regulation of shorter titin isoforms; reduced sarcomere length is maintained throughout adulthood in a large proportion of cardiomyocytes.

### 3.6 Correct sarcomere length can be restored with exogenous Thymosin β4

**Fig. 8:**
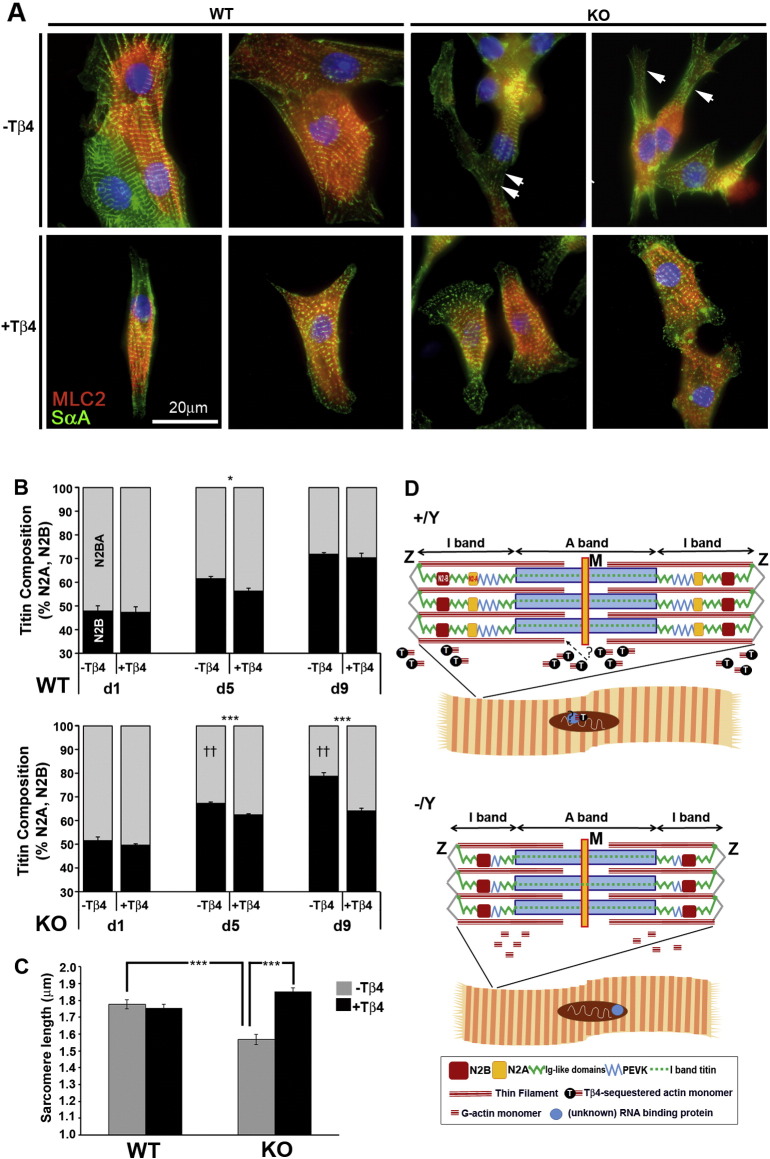
Appropriate sarcomere length can be restored with exogenous Thymosin β4. The N2BA to N2B titin postnatal isoform switch can be recapitulated in cultured cardiomyocytes from E18.5 mouse hearts. A: Wild type (WT) cardiomyocytes displayed an organised sarcomere whereas those from Tβ4 knockout (KO) hearts were shorter in length and partly disordered. Supplementation of culture medium with 100 ng/ml synthetic Tβ4 restored normal morphology and sarcomere organisation in KO cardiomyocytes. Rescue coincided with restoration of normal titin splicing (B; p < 0.01 + Tβ4 vs –Tβ4 in KO cardiomyocytes) and sarcomere length (C), by day 9 in culture. A summary schematic (D) to illustrate the shortened titin and sarcomere/TFL in Tβ4 −/Y cardiomyocytes. The precise mechanism by which Tβ4 impacts titin splicing and/or TFL remains unclear; two possibilities are depicted i) a direct effect on filament extension by regulating the availability of G-actin monomers or ii) a direct effect of titin splicing, via incorporation into splicing complexes, which may also include monomeric nuclear actin. Scale bar in A: 20 μm. *: p < 0.05; ***: p < 0.001, + Tβ4 vs –Tβ4, or as indicated by lines. ††: p < 0.01, KO vs WT. n = 6 per genotype from 3 independent experiments. Error bars in B, C: SEM.

The developmental isoform transition can be accurately recapitulated in cultured cardiomyocytes from late embryonic hearts. Primary cardiomyocyte culture allows for direct manipulation of titin splicing by growth factors or mechanical parameters, as demonstrated using thyroid hormone in rat cardiomyocytes [48]. In order to confirm that short sarcomere length and altered titin splicing were a direct consequence of Tβ4 loss and not secondary to another effect, such as coronary vascular insufficiency, we sought to rescue −/Y sarcomere growth via supplementation of culture medium with synthetic Tβ4. We found that E18.5 murine cardiomyocytes displayed the same N2BA-N2A transition reported for rat [48] (Fig. 8). Wild type (WT) cardiomyocytes displayed an organised sarcomere (Fig. 8A) and synchronous contractility. Cardiomyocytes from Tβ4 knockout (KO) hearts, while contractile, were disproportionally elongated and narrower in shape compared with WT, with excessive branching and sarcomeres which were shorter in length (Fig. 8A) and severely disrupted in places (arrowheads, Fig. 8A). The shorter sarcomere length in KO cardiomyocytes coincided with a higher proportion of N2B titin from day 5 of culture, reaching levels that were 22.7% higher than WT by day 9 (Fig. 8B; p < 0.001; n = 6 from 3 independent experiments). Culturing in the presence of 100 ng/ml Tβ4 resulted in KO cardiomyocytes that were correctly proportioned, comparable in length and width to WT cardiomyocytes with organised sarcomere structure. Sarcomere length was restored to control level (Fig. 8A and C, p < 0.001) and this coincided with restoration of normal titin splicing by day 9 in culture (Fig. 8B; p < 0.01 + Tβ4 vs –Tβ4 in KO cardiomyocytes). In cardiomyocytes, Tβ4 exists throughout the cytoplasm and the nucleus and binds both G-actin and actin filaments (Fig. A3A, B), as previously reported [50]. Interestingly, upon addition of exogenous Tβ4, either to WT or KO cardiomyocytes, Tβ4 bound filamentous actin and, strikingly, accumulated in and around the nucleus (Fig. A3C, D and G, H, respectively). Tβ4 treatment reportedly alters gene expression [51] but there is no insight into the mechanisms via which this is achieved. While the effects of Tβ4 to rescue sarcomere growth and maturation may be mediated via thin filament extension, a direct role in titin splicing cannot be excluded. Indeed, a number of splicing factors were found to be differentially expressed in postnatal −/Y hearts; by qRT-PCR, the serine/arginine-rich splicing factors (SRSFs) 1, 2, 3 and 7 and SFPQ were up-regulated whereas SRSF5 was down-regulated at P21 (Fig. A4, n = 3), consistent with altered splicing in the absence of Tβ4. Further studies are now required to delineate the mechanisms by which Tβ4 influences sarcomere length and/or splicing of genes, including titin.

## 4 Discussion

Sarcomere assembly is a highly orchestrated process, which requires dynamic adaptation in order to accommodate hypertrophic cardiac growth during perinatal development; sarcomeric proteins undergo isoform switching from foetal to adult type during this period to adjust ventricular filling [7]. We reveal that mice lacking the actin monomer binding protein, Tβ4, fail to appropriately regulate sarcomere length, due to a precocious titin isoform transition postnatally and predominance of the less compliant isoforms through to adulthood. Dysregulated sarcomere length manifests as reduced ventricular volumes, diminished stroke volume and a dependence upon raising heart rate and force of contraction for maintenance of a near normal cardiac output. Under conditions of altered cardiac demand, Tβ4 −/Y mice were limited in their ability to increase heart rate and ejection fraction in response to the β1-adrenergic receptor agonist dobutamine. As predicted, −/Y mice were unable to alter stroke volume when treated with the β1-selective antagonist Esmolol, resulting in a further reduction of cardiac output. Thus, despite normal function at rest, Tβ4 −/Y hearts may be compromised in their ability to adapt under (patho-)physiological conditions. A previous study reported that Tβ4 was dispensable for cardiac development and that cardiac function was unaffected in knockout mice [19]; although left ventricular internal diastolic dimensions were shown to be reduced, consistent with the phenotype reported herein, the sarcomeric defects and compensated cardiac function were not explored. We found the short sarcomere phenotype to be present also in skeletal muscle, suggesting a role for Tβ4 in thin filament regulation or splicing more broadly, rather than as a cardiac-specific regulator of splicing. These findings, along with our demonstration of aberrant postnatal titin splicing transition in vitro, may suggest dysregulated sarcomere length as a primary effect, rather than an adaptive response to compensate for altered cardiac function.

Thin filament length is controlled cooperatively by a number of molecular regulators. Titin, as explored in this study, has a primary role but is, by no means, the only player. Tmod1, the cardiac member of the Tropomodulin family, binds tropomyosin and actin filament pointed-end capping proteins to regulate thin filament length [52], [53]. Tight capping of the slow-growing pointed ends by Tmod was believed to maintain uniform TFL, however, sarcomeres have since been shown to be dynamic, with TFL shortening [54] and elongation [55] possible, despite the presence of capping proteins. That said, the importance of Tmod in protecting the pointed end, at least in skeletal muscle, was confirmed with the recent demonstration that thin filament elongation in dystrophic muscle depends upon proteolysis of Tmod to expose free pointed ends [55]. At the fast-growing (barbed) ends, the thin filament is capped by CapZ to retard actin filament assembly [56]. CapZ was proposed to play a role in the dynamic TFL adaptation that occurs in cardiomyocytes in response to mechanical stimulation, as a surrogate for the effects of exercise/hypertrophy on cardiac muscle [57]. The giant F-actin binding protein, Nebulin, spans the length of the thin filament, its C-terminus binding the Z-disc and its N-terminus binding near the pointed end [58]. Nebulin was postulated to act as a thin filament “ruler” (discussed in [59] and [60]), however, the notion was subsequently challenged with the demonstration that nebulin does not extend to the pointed ends, where Tmod caps thin filaments [32]. The role of nebulin, rather, appears to be to stabilize a large core region of the thin filament and generate uniform TFLs in skeletal muscle [34], whereas nebulette, a smaller nebulin homologue in cardiac muscle, stabilizes a comparatively shorter core region to allow individual TFLs to vary according to working SLs in the heart [34]. Although TFL in Tβ4 −/Y hearts correlated with shorter titin isoform expression, the potential influence via other regulators would be worthy of future investigation.

In reconciling the requirement for Tβ4 at the level of sarcomere growth during development, it is a significant challenge to distinguish whether the primary defect is in thin filament extension or in regulation of titin splicing, events which are intimately linked (Fig. 8D). While an alteration in actin filament formation may, at first, seem more likely, given the recognized function of Tβ4 in G-actin sequestration, titin has been regarded as one of the molecular “rulers” that dictates half sarcomere length and mature thin filaments extend according to the size of the predominant titin isoform; consistent with this are the observations that titin is present during the earliest stages of premyofibril assembly [14]. β-Thymosins act as actin buffering molecules to maintain high monomeric actin concentrations, even though intracellular ionic conditions favour its complete polymerization to F-actin [61]. Profilin-dependent dissociation of G-actin-Tβ4 complexes liberates actin for filament assembly [62]. Thus polymerization is not achieved by regulating the actin sequestering activity of Tβ4 itself; rather by actin desequestering agents, under the regulation of upstream signalling [63]. Indeed, in support of this, a recent study documented that over-expressing profilin led to sarcomere elongation, myocyte hypertrophy and impaired function [64]. In Tβ4 −/Y mice, thin filaments are not impaired in their formation and should, therefore, be able to elongate via the same mechanisms. Other G-actin binding proteins presumably compensate for the lack of Tβ4, at least for early sarcomere assembly. Whether a depleted Tβ4-buffered monomer pool limits sarcomere remodelling during developmental cardiac growth remains unresolved but, on balance, disruption of actin filament formation does not appear to underpin the mutant sarcomere phenotype.

An alternative explanation, worthy of future mechanistic exploration, is that splicing of titin is directly regulated by Tβ4. Tβ4 translocates to the nucleus and may function to sequester nuclear actin, a key component of splicing complexes [65]. In support of this, a putative role in pre-mRNA splicing has also been proposed for profilin [66], which functions, like Tβ4, to sequester actin monomers and our data reveal altered expression of splicing factors in Tβ4 −/Y mutants, which may be an attempt to compensate for altered function. In addition, the cellular localisation and activity of splicing factors of the SRSF family is regulated by Akt-mediated phosphorylation and the developmental titin isoform transition has been shown to be principally regulated via phosphatidylinositol 3-kinase/Akt-dependent signalling [48]. Tβ4 forms a functional complex with PINCH and integrin-linked kinase, to regulate Akt activation [67] and this intersection with the splicing machinery may alternatively explain the requirement for Tβ4 for correct titin splicing.

Processing of titin pre-mRNA is subject to subtle regulatory mechanisms that control entry to either N2B or N2BA splice pathways. RBM20, a member of the SRSF family, regulates the splicing of many genes that are linked to cardiomyopathy, including titin [25]. A loss-of-function mutation in human RBM20, when modelled in rats, results in a DCM-like phenotype due to persistent expression of a giant foetal titin isoform (N2BA-G) in adult heart. Whilst RBM20 expression was not significantly altered in Tβ4 −/Y postnatal hearts, its function may be adversely affected. It remains to be determined whether RBM20, or a related SRSF, could also direct the splicing machinery towards enhanced production of the N2B isoform and dysregulated sarcomere length.

The following are the Supplementary data related to this article.

Video 1 High temporal resolution cine-MRI to assess global functional and morphological parameters in +/Y (WT) and −/Y (KO) mice. Significantly reduced left ventricular end-diastolic volumes in −/Y mice are apparent, shown here in mid-ventricular short axis (KO1, middle panel, compared with WT, left panel). Distinct regional variability in % fractional wall thickening was apparent in −/Y hearts, consistent with the patchy distribution of cardiomyocytes with shorter sarcomeres. Variability in wall motion contributed to a more pronounced twisting motion in −/Y hearts upon contracion (particularly evident in KO2, right panel). Video 1

Video 2 Tβ4 knockout mice subjected to dobutamine stress testing, with high temporal cine MR imaging. The inotropic effects of dobutamine increase cardiac output via elevated heart rate and enhanced myocardial contractility. +/Y control mice (WT) decreased ESV with dobutamine treatment, as evidence of a normal contractile reserve. In contrast, −/Y (KO) mice already display small ESV at baseline, and this is only modestly reduced by dobutamine treatment. This test reveals that Tβ4 −/Y mice possess a limited contractile reserve. Video 2

Video 3 Tβ4 -/Y mice depend upon increasing heart rate and ejection fraction to maintain an effectively normal cardiac output. Bolus infusion of the β1-selective antagonist esmolol slowed heart rate and increased LV volumes in both +/Y (WT) and −/Y (KO) hearts. Whereas esmolol treatment markedly reduced SV in WT mice, KO mice were unaffected; cardiac output dropped significantly in KO mice. Video 3

Supplementary material Image 1

## Disclosures

The authors have no conflict of interest to declare.
