# Supplementary figures and images for: Aberrant developmental titin splicing and dysregulated sarcomere length in Thymosin β4 knockout mice
Source: J Mol Cell Cardiol. 2017 Jan;102:94–107. doi: 10.1016/j.yjmcc.2016.10.010 (PMC5319848; doi:10.1016/j.yjmcc.2016.10.010)

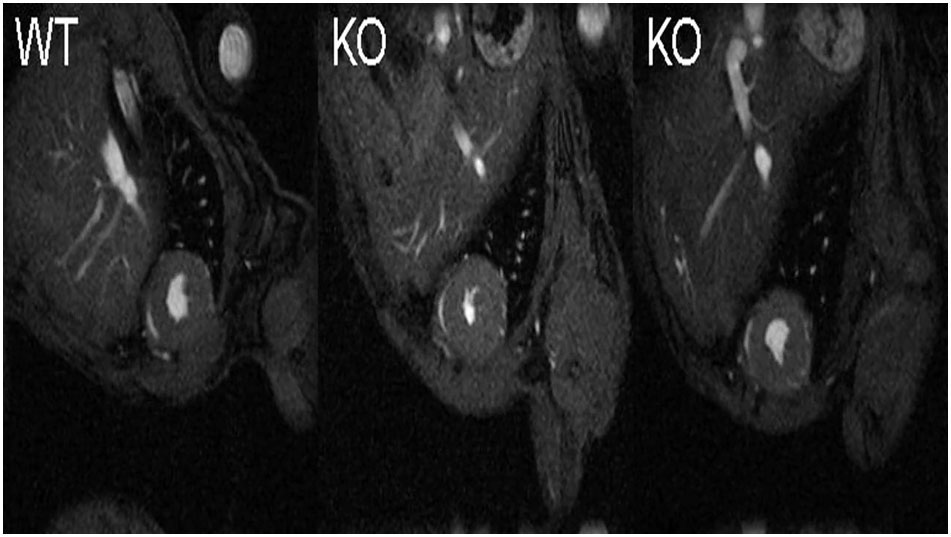

Supplement: Video 1 — High temporal resolution cine-MRI to assess global functional and morphological parameters in +/Y (WT) and −/Y (KO) mice. Significantly reduced left ventricular end-diastolic volumes in −/Y mice are apparent, shown here in mid-ventricular short axis (KO1, middle panel, compared with WT, left panel). Distinct regional variability in % fractional wall thickening was apparent in −/Y hearts, consistent with the patchy distribution of cardiomyocytes with shorter sarcomeres. Variability in wall motion contributed to a more pronounced twisting motion in −/Y hearts upon contracion (particularly evident in KO2, right panel). [file mmc1.jpg]

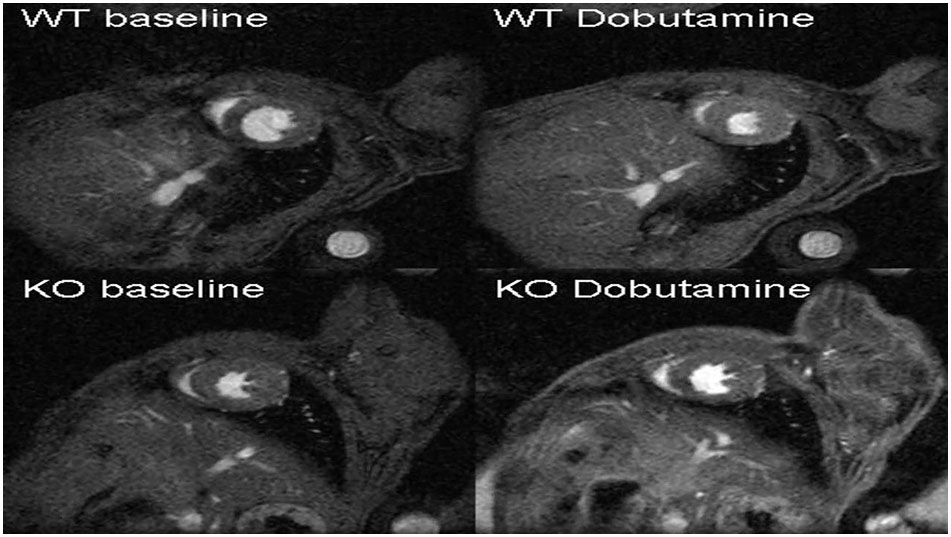

Supplement: Video 2 — Tβ4 knockout mice subjected to dobutamine stress testing, with high temporal cine MR imaging. The inotropic effects of dobutamine increase cardiac output via elevated heart rate and enhanced myocardial contractility. +/Y control mice (WT) decreased ESV with dobutamine treatment, as evidence of a normal contractile reserve. In contrast, −/Y (KO) mice already display small ESV at baseline, and this is only modestly reduced by dobutamine treatment. This test reveals that Tβ4 −/Y mice possess a limited contractile reserve. [file mmc2.jpg]

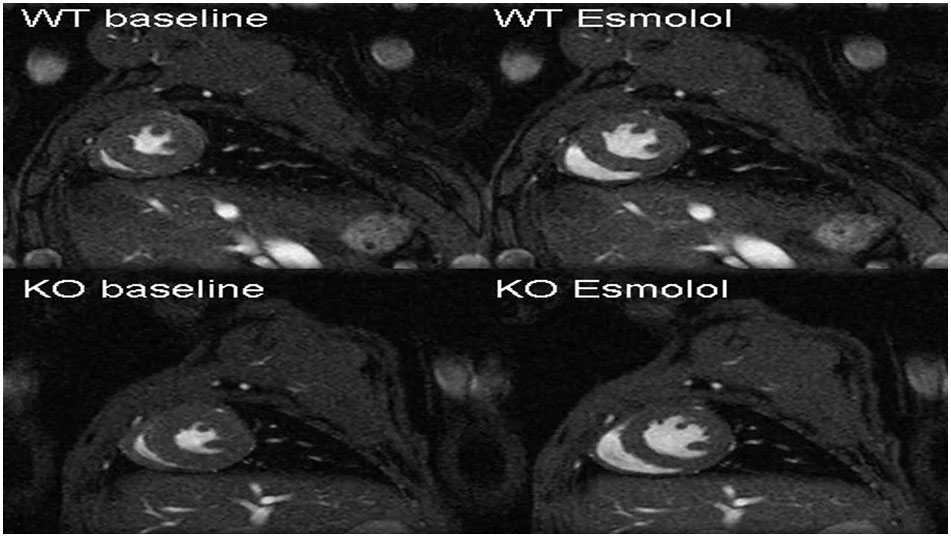

Supplement: Video 3 — Tβ4 -/Y mice depend upon increasing heart rate and ejection fraction to maintain an effectively normal cardiac output. Bolus infusion of the β1-selective antagonist esmolol slowed heart rate and increased LV volumes in both +/Y (WT) and −/Y (KO) hearts. Whereas esmolol treatment markedly reduced SV in WT mice, KO mice were unaffected; cardiac output dropped significantly in KO mice. [file mmc3.jpg]
