# Supplementary material for: Aberrant developmental titin splicing and dysregulated sarcomere length in Thymosin β4 knockout mice
Source: J Mol Cell Cardiol. 2017 Jan;102:94–107. doi: 10.1016/j.yjmcc.2016.10.010 (PMC5319848; doi:10.1016/j.yjmcc.2016.10.010)
Supplement: Supplementary file 4 — Supplementary material [file mmc4.pdf]

Aberrant developmental titin splicing and dysregulated sarcomere length in Thymosin β4 knockout mice

Nicola Smart[a], Johannes Riegler[b,c], Cameron W. Turtle[d], Craig A. Lygate[d], Debra J. McAndrew[d], Katja Gehmlich[d], Karina N. Dubé[e], Anthony N. Price[b,f], Vivek Muthurangu[g], Andrew M. Taylor[g], Mark F. Lythgoe[b], Charles Redwood[d] and Paul R. Riley[a]

[a]Department of Physiology, Anatomy and Genetics, University of Oxford, Oxford, UK; [b]Centre for Advanced Biomedical Imaging, Department of Medicine, University College London (UCL), London, UK; [c]Genentech Inc., 1 DNA Way, South San Francisco, California 94080, USA; [d]Division of Cardiovascular Medicine, Radcliffe Department of Medicine, University of Oxford, Oxford, UK; [e]UCL-Institute of Child Health, London UK; [f]Department of Perinatal Imaging & Health, King's College London, UK; [g]Centre for Cardiovascular Imaging, UCL Institute of Cardiovascular Science, London, UK.

Corresponding author: Dr Nicola Smart
E-mail: nicola.smart@dpag.ox.ac.uk

ONLINE SUPPLEMENTARY MATERIAL

Table A1. Cardiac functional analysis by cine MRI

	+/Y (n=14)	-/Y (n=16)	p value*
**Body weight, g**	32.7 ± 1.0	31.1 ± 0.9	n.s.
**Heart rate, bpm**	519 ± 14	540 ± 10	n.s.
**Age, days**	130 ± 6	132 ± 7	n.s.
**EDV, µl**	67.7 ± 1.8	57.4 ± 2.2	< 0.01
**ESV, µl**	23.2 ± 1.2	16.9 ± 1.5	< 0.01
**SV, µl**	44.5 ± 1.4	40.4 ± 1.0	< 0.05
**EF, %**	66 ± 1	71 ± 2	< 0.05
**CO, ml/min**	23.1 ± 0.9	21.8 ± 0.6	n.s.
**LVM, mg**	106.0 ± 1.8	102.2 ± 2.6	n.s.

* two-tailed unpaired Student's t-test

Table A2: PV Haemodynamic data

	+/Y (n=4)			-/Y (n=6)			p value*		
	Baseline	Dobutamine	Δ Baseline	Baseline	Dobutamine	Δ Baseline	Baseline	Dobutamine	Δ Baseline
**Corrected ESP (mmHg)**	91.1 ± 4.2	89.1 ± 4.6	-2.1 ± 2.3	95.5 ± 2.8	93.3 ± 3.3	-2.2 ± 2.8	1	0.201	1
**Corrected EDP (mmHg)**	5.0 ± 1.7	5.4 ± 1.5	0.4 ± 1.6	4.6 ± 1.7	5.8 ± 2.1	1.2 ± 0.8	0.670	0.286	0.762
**Heart Rate (BPM)**	467.1 ± 44.9	539.2 ± 38.2	72.1 ± 19.7	493.0 ± 25.2	557.9 ± 34.4	64.9 ± 14.1	0.522	0.394	0.61
**Max dP/dt (mmHg/s)**	8643.2 ± 660.1	11300.8 ± 1172.4	2657.6 ± 654.4	9798.3 ± 751.5	11210.47 ± 1045.64	1412.2 ± 758.5	0.054	0.831	0.114
**Min dP/dt (mmHg/s)**	-6689.9 ± 1213.9	-6895.8 ± 1390.0	-205.9 ± 542.8	-7992.1 ± 376.1	-8001.2 ± 432.0	-9.1 ± 393.3	0.286	0.522	0.352
**Tau (ms)**	7.40 ± 1.0	6.48 ± 1.0	-0.92 ± 0.4	6.4 ± 0.8	5.9 ± 0.7	-0.5 ± 0.5	0.088	1	0.114

Means ± SD

* Exact significance, Mann-Whitney test

Table A3. Left Ventricular Segmentation<br>Segment	p value
1. basal anterior	0.54
2. basal anteroseptal	0.03*
3. basal inferoseptal	0.93
4. basal inferior	0.43
5. basal inferolateral	0.12
6. basal anterolateral	0.52
7. mid anterior	0.04*
8. mid anteroseptal	0.19
9. mid inferoseptal	0.8
10. mid inferior	0.53
11. mid inferolateral	0.22
12. mid anterolateral	0.02*
13. apical anterior	0.16
14. apical septal	0.04*
15. apical inferior	0.39
16. apical lateral	0.37

F test to compare variances of +/Y vs -/Y

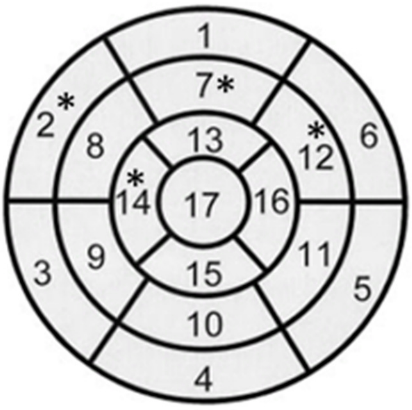

Table A4: Left ventricular functional indices during Dobutamine stress

	+/Y (n=4)			-/Y (n=6)			p value*
	Baseline	Dobutamine	Δ Baseline	Baseline	Dobutamine	Δ Baseline	
heart rate [bpm]	505±58	549±23	87 ± 58	549±23	584±7	35 ± 25	0.082
EDV (slice) [µl]	9.85±0.77	8.22±1.14	-1.64±0.95	10.03±1.69	8.76±1.31	-1.24 ± 0.76	0.730
ESV (slice) [µl]	2.84±0.75	0.79±0.43	-2.05±0.48	1.84±1.04	0.85±0.49	-0.98 ± 0.69	0.114
EF [%]	71.4±5.42	90.71±4.08	19.31±3.06	81.87±8.22	90.02±5.07	8.15 ±5.78	0.029
PER [ml/sec]	-0.25±0.03	-0.30±0.04	-0.06±0.02	-0.32±0.06	-0.34±0.04	-0.02 ± 0.04	0.200
PFR [ml/sec]	0.28±0.03	0.30±0.03	0.02±0.02	0.28±0.03	0.32±0.09	0.04 ± 0.07	0.730
Mean EDWT [mm]	0.98±0.10	1.07±0.18	0.09±0.09	1.02±0.09	1.07±0.07	0.04 ± 0.04	0.556
Mean ESWT [mm]	1.45±0.18	2.01±0.20	0.56±0.26	1.81±0.33	2.03±0.19	0.22 ± 0.17	0.114

Means ± SD

PER: Peak Ejection Rate; PFR: Peak Filling Rate; EDWT: End-diastolic wall thickness; ESWT: End-systolic wall thickness.

* Exact significance, Mann-Whitney test, Δ Baseline +/Y vs -/Y

Table A5: Left ventricular function during Esmolol treatment

	+/Y (n=9)			-/Y (n=9)			p value		
	Baseline	Esmolol	Δ Baseline	Baseline	Esmolol	Δ Baseline	Baseline‡	Esmolol‡	Δ Baseline*
heart rate [bpm]	527 ± 54	460 ± 41	-67 ± 10	536 ± 49	446 ± 23	-91 ± 12	1	1	0.11
EDV [µl]	71.3 ± 3.5	84.9 ± 6.6	13.7 ± 1.7	60.0 ± 9.4	75.1 ± 4.1	15.2 ± 2.3	0.015	0.004	0.73
ESV [µl]	24.0 ± 4.9	39.9 ±7.6	15.8 ± 1.8	18.3 ± 7.3	33.4 ± 3.3	15.2 ± 1.7	0.182	0.304	0.86
SV [µl]	47.2 ± 3.5	45.1 ± 3.3	-2.1 ± 0.8	41.7 ± 2.7	41.7 ± 1.4	0.0 ± 0.9†	0.109	0.002	0.05
EF [%]	66 ± 6	53 ± 6	-13 ± 1	71 ± 8	56 ± 2	-15 ± 2	1	0.796	0.6
CO [ml/min]	24.8 ± 2.1	20.7 ± 1.3	-4.1 ± 0.6	22.2 ± 1.9	18.6 ± 1.2	-3.6 ± 0.4	0.071	0.013	0.26

Means ± SD

‡ ANOVA with Bonferroni correction, +/Y vs -/Y
† By ANOVA (Bonferroni correction), Baseline vs Esmolol was $p<0.05$ for all +/Y and -/Y parameters, except SV
* Exact significance, Mann-Whitney test, Δ Baseline +/Y vs -/Y

Table A6: Echocardiographic Measurements

	+/Y (n=3)		-/Y (n=6)		
	Mean	SD	Mean	SD	p value*
A'	-12.90	1.05	-10.96	2.55	0.71
E'	-19.36	1.08	-17.52	3.69	0.91
MV A	342.88	94.90	426.39	65.42	0.38
MV E	617.63	160.21	777.26	54.44	0.17
A'/E'	0.67	0.05	0.63	0.11	1.00
E'/A'	1.50	0.11	1.63	0.33	1.00
MV E/A	1.81	0.14	1.87	0.42	0.71
MV E/E'	-32.12	9.28	-45.43	16.76	0.17

Means ± SD

* Exact significance, Mann-Whitney test

Extended Materials and Methods

Global Tβ4 KO mice

As previously described [26], the Tβ4 gene targeting vector was created by ligating a 6.5kb fragment containing a 5'arm (exon 1; BamHI-EcoRV) and 3' arm (exon 3; Nhe1-BamH1) flanking a neomycin resistance cassette, thereby deleting exon 2 of the *Tmsb4x* gene. The construct was linearised and electroporated into ES cells which were subject to positive-negative selection and screening by Southern blot analysis for homologous recombination using 5' flanking and internal probes. Male chimaeras were generated from targeted ES cell lines and F1 mice generated by crossing to wild type C57Bl6/J females. Progeny were genotyped using a PCR that amplifies both mutant and wild type alleles. Mice have been maintained on a C57Bl6/J background for more than 25 generations. Experiments were performed on 14-19 week old mice except, as indicated, for developmental time course data.

MR Image acquisition

Mice were anaesthetised with isoflurane (4%) and placed onto an animal cradle in supine position. Animals were kept at 37±1 °C (during image acquisition) via a water- and air- heating system while oxygen and anaesthetics (1-2% isoflurane) were supplied via a nose cone (1 L/min). A neonatal apnoea sensor was placed on the abdomen for respiratory gating. Electrocardiogram (ECG) was obtained by needle electrodes inserted subcutaneously into the forelimbs. For Dobutamine and Esmolol stress tests an intraperitoneal (i.p.) infusion line was prepared and connected to an infusion pump (PHP2000 Harvard Instruments, UK). Image acquisition was performed with a 39 mm diameter volume coil (Rapid Biomedical GmbH, Germany). Cardio-respiratory monitoring and gating were performed using an MR-compatible system (SA Instruments, NY).

Imaging was performed using a 9.4T VNMRS horizontal bore scanner (Agilent Technologies, CA) with a shielded gradient system (1000 mT/m). Long and short axis scout images were acquired to define the two and four chamber long axis view. The cine long axis views were used to define the short axis orientation. An ECG and respiratory gated spoiled gradient echo (GE) sequence was used to acquire cine cardiac images with the following parameters for standard cine acquisitions: TE 1.18 ms, TR 4.5 ms, flip angle 15°, slice thickness 1 mm, no slice separation, FOV 25.6×25.6 mm², matrix size 128×128, NSA 2. Twenty cine-frames were recorded to cover the cardiac cycle. A single short-axis slice was obtained in approximately 55 seconds, leading to a total scan time of around 9 to 11 minutes covering the apex to base (10-12 slices).

In order to assess the filling and discharging pattern of the left ventricle at baseline or under Dobutamine stress, a mid-ventricular short-axis slice was selected and images were acquired with a short TR using the following imaging parameters: TE 1.07 ms, TR 2.4 ms, flip angle 10°, slice thickness 1 mm, FOV 25.6×25.6 mm², matrix size 128×128, NSA 2. Sixty-two cine-frames were recorded to cover approximately 1.5 cardiac cycles. After the acquisition of the baseline images, i.p. infusion of Dobutamine was started at 40 μg kg^{-1} min^{-1}. Image acquisition under Dobutamine stress was started after the heart rate had increased by approximately 10% (10-15 minutes).

In order to assess the effect of the β-blocker Esmolol, a full stack of baseline cine images were acquired before i.p. Esmolol infusion was started at 5 mg kg^{-1} min^{-1}. A reduction in heart rate by approximately 80 beats per minute within 5-7 minutes was observed. When the heart rate had stabilised, a full cine stack, including long axis views, was acquired using the same imaging parameters as for standard cine (see above).

MR Image analysis

Short and long axis images for each animal were combined into one dataset, randomised and anonymised. Images were analysed as previously described [27], using the cardiac analysis software Segment (http://segment.heiberg.se). Briefly, short axis images were automatically segmented with the LV tool of Segment followed by manual adjustments where necessary. Papillary muscles were segmented as circular objects surrounded by blood on short-axis slices at end-diastole and treated as myocardium. When papillary muscles were pressed against the endocardial wall at end-systole, they were segmented as myocardium by including them within the endocardial border, as previously

described [27]. End diastolic volume (EDV) and end systolic volume (ESV) were identified by the global maximum and minimum LV cavity volume respectively. The left ventricular mass (LVM) was calculated as the difference between epicardial and endocardial volume at end diastole multiplied by 1.05 g/ml. Other parameters were calculated as follows: Stroke volume (SV) = EDV – ESV; Ejection fraction (EF) = 100 * SV / EDV; Cardiac output (CO) = SV * heart rate. Papillary muscles were treated as myocardium for all volume measurements, except when generating Bulls eye plots where papillary muscles were treated as part of the blood pool. The Bulls eye plots were generated with Segment following the AHA convention.

The mid-ventricular cine images with higher temporal resolution for rest and Dobutamine stress were randomised and anonymised. Images were automatically segmented using Segment with manual corrections for papillary muscles if necessary. Peak ejection rate (PER) and peak filling rate (PFR) were calculated from the maximum change in blood volume (dV/dt) using a moving average over two values.

Echocardiographic and haemodynamic assessment of function

Echocardiograms were obtained in male +/Y and -/Y mice at 17-18 weeks of age by a single operator. Mice were anaesthetised with 4 % isoflurane in medical oxygen and maintained at 1–1.5 % by nose-cone on a homeothermic platform. Chest fur was shaved for imaging with a Visualsonics Vevo 2100 using a 22–55 MHz transducer. Pulse wave Doppler measurements were obtained across the mitral valve via the apical 4-chamber view and tissue Doppler imaging for radial velocities via a parasternal short-axis view. Haemodynamic measurements were performed on the same mice 3-4 days later under identical anaesthetic conditions. The LV was cannulated via the right carotid artery using a 1.4-F Millar Mikro-Tip cannula (SPR-839, Millar Instruments, Houston, Texas). The right jugular vein was cannulated with stretched polyethylene tubing for infusion of dobutamine hydrochloride (16 μg kg^{-1} min^{-1}) to test contractile reserve. Measurements were obtained after 15 min of equilibration via a Powerlab 4SP data acquisition system (ADInstruments, UK). All data acquisition and analysis was performed blind to genotype.

Force-Ca^{2+} sensitivity measurement

Solution compositions for force-Ca^{2+} sensitivity measurements were determined using an iterative computer program that uses published affinity constants[28] to calculate the equilibrium concentration of ligands and ions. Solutions contain (in mmol L-1): 80 MOPS, 15 EGTA, 1 Mg^{2+}, 5 MgATP, 135 (Na^{+} + K^{+}), 15 creatine phosphate and 20 units ml-1 creatine phosphokinase, pH 7.0 (at 15°C). Ca^{2+} concentration was varied using $CaCl_2$. Total ionic strength was 170 mmol L-1. Mice were killed via cervical dislocation. Hearts were rapidly excised and dissected in oxygenated physiological salt solution. Left ventricular trabeculae (width: 80-250 μm, length: 1.0-3.0 mm) were cut and washed overnight at 4°C in relaxing solution containing 1 % Triton X-100. After skinning, fibres were stored in 50% glycerol relaxing solution at -20 °C for experiments within 1 week. Skinned trabeculae were assessed using an Aurora Scientific Permeabilized Fibre Test System. Sarcomere length was set at 2.0 μm in relaxing solution using laser diffraction and experiments were conducted at 15 °C. Isometric force was measured by transiently shortening the fibre to slack length. Passive force, measured in relaxing solution, was subtracted from total force at each Ca^{2+} concentration to determine active force.

Adult Cardiomyocyte isolation

Murine left ventricular myocytes were isolated using an enzymatic digestion technique. The heart was submitted to retrograde perfusion with a Ca^{2+}-free buffer for 3 minutes followed by a 1mg/ml Collagenase type II solution (250 units/mg, Worthington Biochemical Corporation) for 9 minutes. The digested tissue was mechanically agitated in enzymatic solution for an additional 5 minutes and the cells were collected by centrifugation at 500 rpm.

Functional assessment of cardiomyocytes

Functional assessment of the isolated cardiomyocytes was conducted within 6 hours of isolation using an IonOptix-μStep Myocyte Contractility System. Fractional shortening was assessed via Fast Fourier Transform-based sarcomere length tracking. [Ca^{2+}] transients were measured in cells loaded with 1mM Fura-2-AM (Life Technologies) and 2μM Pluronic.

Immunohistochemistry and sarcomere length determination
Imaging of fixed cardiomyocytes was performed using a Leica TCS SP5 II system. Leica Application Suite (LAS) AF Lite was used to measure image intensity over a line segment drawn perpendicular to visible striations. Matlab was used to locate peaks in the intensity plot and calculate the associated difference between peaks. The mean difference between peaks for each cell was considered its sarcomere length.

Embryonic Cardiomyocyte Culture
Primary cultures of cardiomyocytes were prepared from WT or Tβ4 KO E18.5 mouse hearts, essentially as previously described for neonatal cardiomyocytes[29], and cultured at an initial density of 1×10^6 cells per well in a 6-well plate (with gelatin-coated coverslips for immunofluorescence analysis). Cardiomyocytes were >95% pure at time of plating as determined by immunofluorescence detection of cardiac sarcomeric markers. After overnight culture in plating medium, cells were washed twice in PBS and medium replaced with maintenance medium (4:1 DMEM:M199; 1% FBS) containing either PBS (vehicle control) or Tβ4 (100ng/ml, a kind gift from RegeneRx Pharmaceuticals). Medium, supplemented with fresh Tβ4 or PBS, was changed daily. Cells were harvested after 1, 5 and 9 days for real time qPCR and immunofluorescence analysis.

Immunodetection Methods
Immunofluorescence was performed on cryosections of diastole-fixed mouse hearts or on soleus or tibialis anterior muscle from mice at various developmental stages and on embryonic murine cardiomyocytes using standard protocols with the following antibodies: sarcomeric α-actinin (SαA; Sigma), Thymosin β4 (Immundiagnostik), myomesin and cardiac myosin binding protein C (cMyBPC a kind gift of Elisabeth Ehler/ Mathias Gautel), cTnT and cTNI, (Abcam); N2B, PEVK or N2A (kind gifts of Siegfried Labeit). Images were acquired using either an Olympus IX81 microscope, a Zeiss AxioImager with ApoTome or a Leica DM6000 fluorescence microscope with Structured Illumination.

Thin Filament and Sarcomere Length Measurements on Sectioned Left Ventricle
Hearts were fixed in diastole by injection of 100mM KCl into the left atrium of mice while anaesthetised with isoflurane. After rapid arrest in diastole, 4% PFA in phosphate buffered saline (PBS) was perfused for 2-3 minutes before hearts were removed and incubated in the same fixative for a further 2 hours. After washing with PBS and embedding in Tissue-Tek O.C.T. compound, hearts were rapidly chilled in isopentane, within a dry ice bath. 7μm thick sections were prepared and stained, as described above. Confocal images were obtained using an Olympus IX81 confocal microscope (6 left ventricular fields of view per section from 3 sections at different levels through the heart, from 5 -/Y and 3 +/Y hearts). Images were analysed in ImageJ (http://rsb.info.nih.gov/ij), with 1D plot profiles drawn along the myofibril, perpendicular to the Z-lines. Well-defined peaks were obtained, as shown in Fig. 2T, in which major peaks represented α-actinin positive Z-lines and minor peaks represented Tmod-1 positive thin filament ends. Sarcomere length (SL) was measured as the distance between 2 α-actinin peaks (at their centres, via a perpendicular line) and TFL as the distance between an α-actinin peak and a Tmod1 peak.

Histological Methods
Sections of adult heart were stained with hematoxylin and eosin, using a standard protocol, or Alexa 594-conjugated wheatgerm agglutinin (Invitrogen), according to the manufacturer's instructions. Nuclear counts, cell area and cell counts were performed on sections which had been anonymised and blinded for genotype, using ImageJ software particle analysis.

Analysis of titin isoforms by SDS-PAGE
Hearts were homogenized in sample buffer containing 8 M urea, 2 M thiourea, 3% SDS, 75 mM DTT, 10% glycerol, bromophenol blue and 0.05 M Tris·HCl, pH 6.8 (samples prepared as described in[23]). Samples were incubated for 5 min on ice and boiled for 5 min at 95°C, followed by centrifugation. Agarose-strengthened SDS-PAGE (2% polyacrylamide; 0.5% agarose) was performed, at 2mA

overnight, and protein bands were visualized, following Coomassie brilliant blue staining, and scanned using a ChemiDoc system (BioRad). Densitometry analysis was performed using ImageJ to determine relative intensities and % total titin.

RNA isolation and gene expression profile by real time qRT-PCR

Total RNA was isolated from the ventricles of collected hearts using Trizol reagent (Invitrogen) or from embryonic murine cardiomyocytes using the RNeasy Plus Micro kit (Qiagen), according to the manufacturer's instructions, and reverse-transcribed using Superscript III RT (Invitrogen). Real-time RT-PCR analysis was performed on an ABI 7900 Sequence Detector using SYBR Green (Applied Biosystems). Data were normalised to *Hprt1* expression (endogenous control). Fold-changes in gene expression were determined by the $2^{-\Delta\Delta CT}$ method [30].

Real time qRT-PCR Primers

N2A	F: GCAAGCACGAAGACCTCGAA
(Ex 107-108)	R: CGAAAGTAGCAGACTGATGCTC
N2B	F: CTGCAAAGCCTCCAATGAGT
(Ex 50-219)	R: TTTCAGCACCACCTCTTCCT
Myh7	F: ACTGTCAACACTAAGAGGGTCA
	R: TTGGATGATTTGATCTTCCAGGG
Nppa	F: TTCCTCGTCTTGGCCTTTTG
	R: CCTCATCTTCTACCGGCATCTTC
Nppb	F: GAGGTCACTCCTATCCTCTGG
	R: GCCATTTCCTCCGACTTTTCTC
Srsf1	F: TCTCACGAGGGAGAAACTGC
	R: CTGCTACGGCTTCTGCTACG
Srsf2	F: CGCGCTCCAGATCAACCTC
	R: CTTGGACTCTCGCTTCGACAC
Srsf3	F: GCGCAGATCCCCAAGAAGG
	R: ATCGGCTACGAGACCTAGAGA
Srsf5	F: AGGACGATACTCCGACCGTTT
	R: GCAAAGGTTACTTCCCCAGC
Srsf6	F: CAAAAATGGGTACGGTTTCGTG
	R: AGTTCTCCGACTGCTGTATCC
Srsf7	F: AGGACGGAGGTCAAGATCAG
	R: GGGGAACGACTTCTTTTAGGAGA
Sf3b1	F: GTGGGCCTTGATTCCACAGG
	R: GGCTTCTTCTGACCGAGCAA
Sfpq	F: TGTCGGTTGTTTGTGGGGAAT
	R: AACCCGAACCCTTTGCCTTT
Rbm20	F: CCTGCCTTTGGGTCTCGGCTTAAC
	R: CCCTTTCACATGTAGCTCCCAGTC
Hprt1	F: TCAGTCAACGGGGGACATAAA
	R: GGGGCTGTACTGCTTAACCAG

Statistical analysis

For the comparison of LV baseline data, a two-tailed unpaired Student's t-test was used to determine any significant differences ($P < 0.05$). The requirements for a t-test were assessed using a Shapiro-Wilk test for normality and an F-Test to compare the variances. For the comparison of functional indices at rest and under Esmolol treatment, a two-way ANOVA with Bonferroni correction was used. Dobutamine MRI, ECHO and haemodynamic data were analysed using a two-tailed Mann–Whitney test with multiple correction. Statistical analysis was performed using R software version 2.8.1 or SPSS 22.

Fig. A1

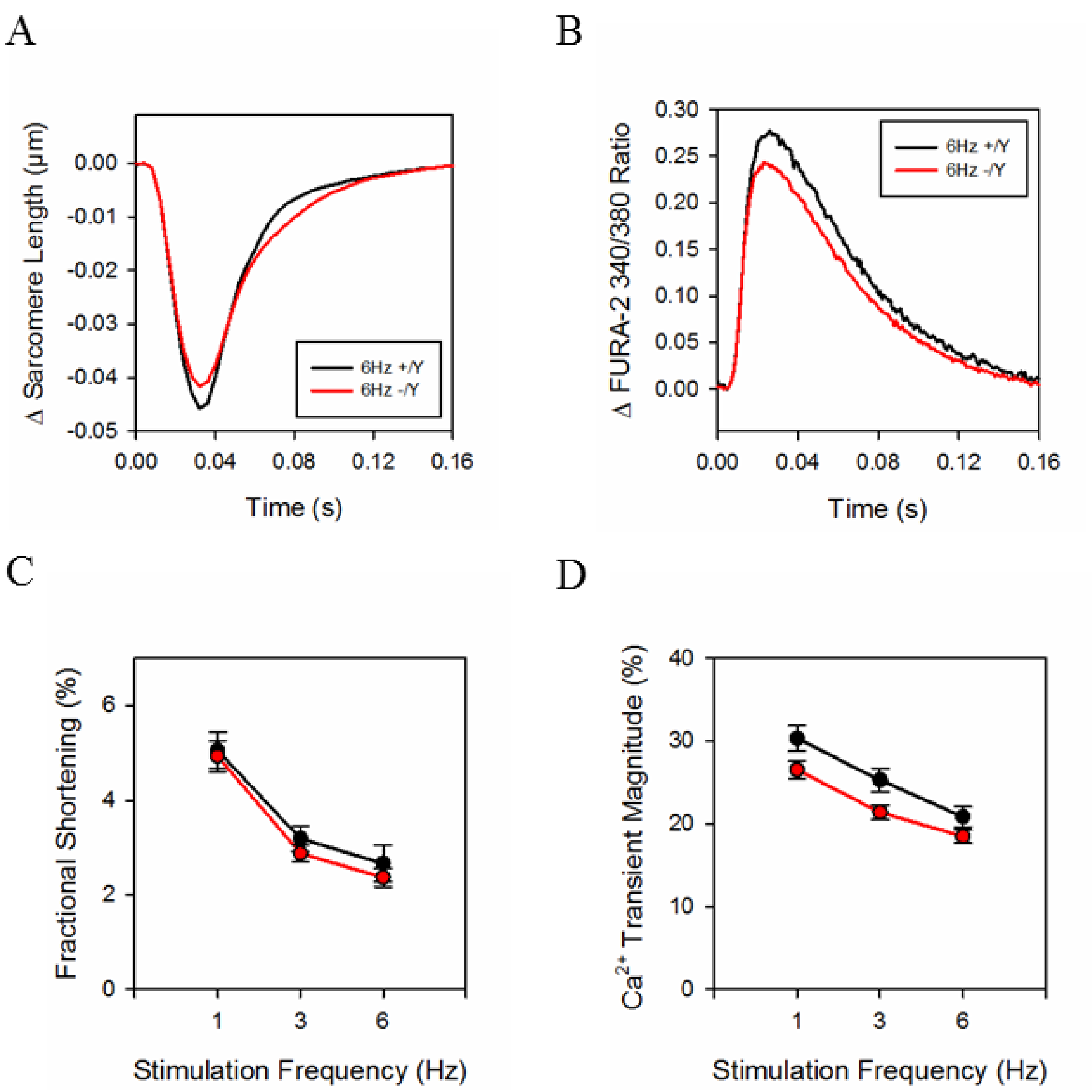

Cardiomyocytes from Tβ4 null mice exhibit no significant differences in shortening or Ca^{2+} handling parameters. Averaged shortening (**A**) and Ca^{2+} transient (**B**) traces at 6Hz stimulation frequency from cardiomyocytes isolated from control (+/Y, black) and Tβ4 null (-/Y, red) mice were broadly similar. No consistent significant differences were detected in fractional shortening (**C**) or Ca^{2+} transient magnitudes (**D**) across all stimulation frequencies. (n=55 +/Y and 63 -/Y; Sarcomere length = 1.76 +/- 0.01µm (+/Y); 1.63 +/- 0.03µm (-/Y; p=0.01).

Fig. A2

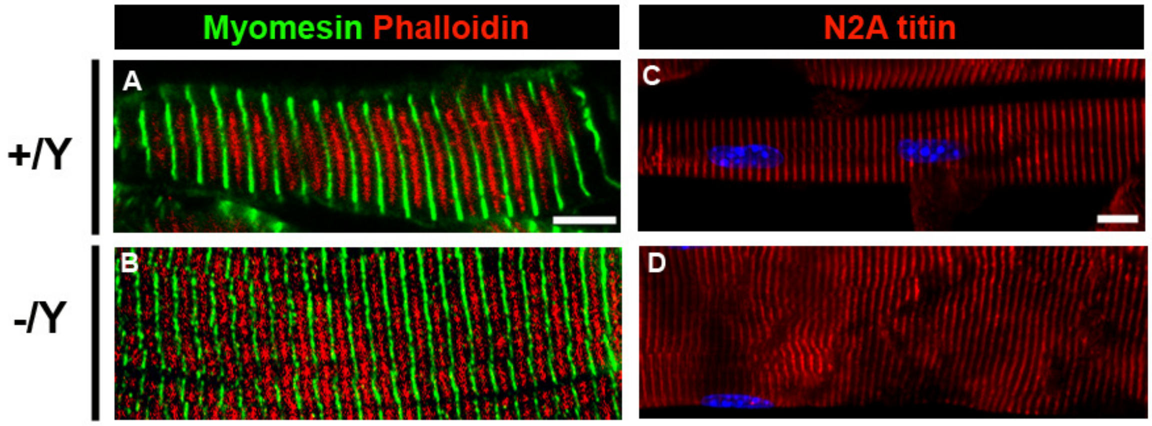

Shortened sarcomere length in skeletal muscle from Tβ4 knockout mice. Representative longitudinal sections of Soleus muscles from Tβ4 -/Y mice (n=5) and +/Y controls (n=3). Closer apposition of Z- and M- bands was revealed with dual myomesin/phalloidin staining (A +/Y vs B -/Y); shortened titin was confirmed by the reduced spacing of the N2A element (C +/Y vs D -/Y).

Fig. A3

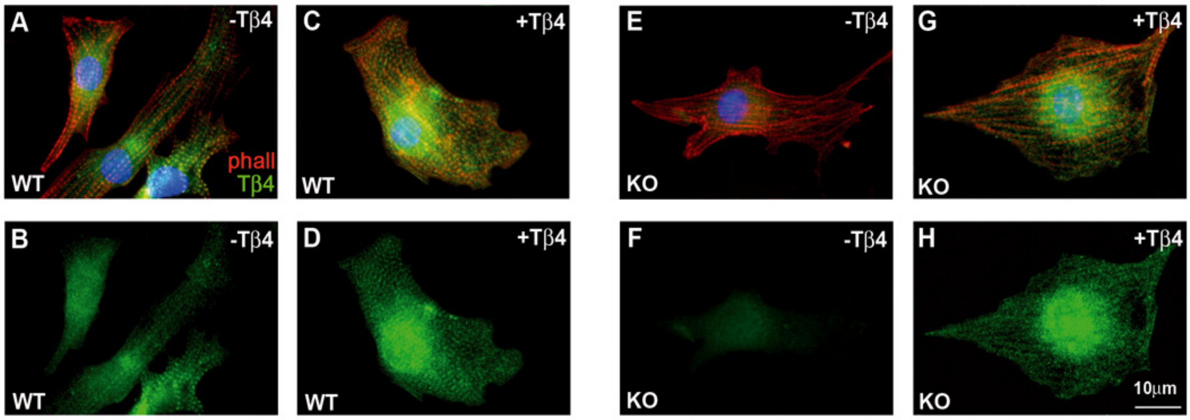

Cellular compartmentalisation of endogenous and exogenous Tβ4. In WT cardiomyocytes, Tβ4 localises throughout the cytoplasm and the nucleus and binds not only to G-actin but also to actin filaments (**A**, showing co-localisation with phalloidin-stained F-actin, and **B**). As expected, no Tβ4 expression is detected in KO cardiomyocytes (**E, F**). Upon addition of exogenous Tβ4, either to WT or KO cardiomyocytes (**C, D** and **G, H**, respectively), Tβ4 binds filamentous actin and strikingly accumulates in and around the nucleus.

Fig. A4

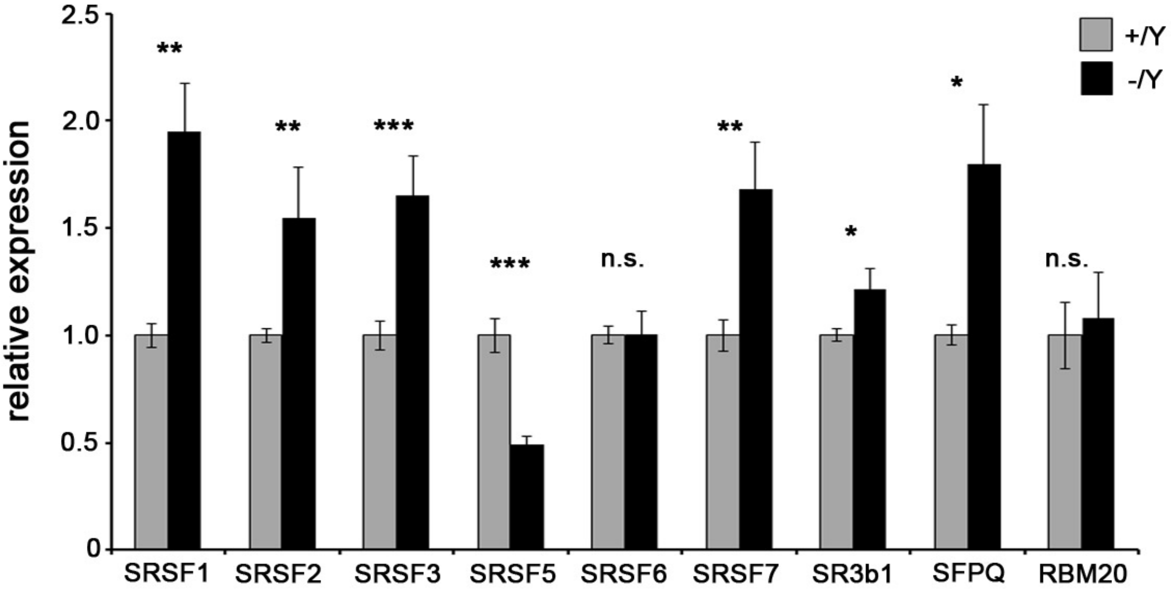

Serine/arginine-rich splicing factors (SRSFs) are mis-regulated in postnatal Tβ4 -/Y hearts. qRT-PCR revealed SRSFs 1, 2, 3 and 7 and SFPQ were up-regulated whereas SRSF5 was down-regulated, in postnatal Tβ4 -/Y hearts, shown at P21. Expression of RBM20 was not significantly altered. *: $p<0.05$; **: $p<0.01$; ***:$p<0.001$.

Legends for Video Files

Video 1

High temporal resolution cine-MRI to assess global functional and morphological parameters in +/Y (WT) and -/Y (KO) mice. Significantly reduced left ventricular end-diastolic volumes in -/Y mice are apparent, shown here in mid-ventricular short axis (KO1, middle panel, compared with WT, left panel). Distinct regional variability in % fractional wall thickening was apparent in -/Y hearts, consistent with the patchy distribution of cardiomyocytes with shorter sarcomeres. Variability in wall motion contributed to a more pronounced twisting motion in -/Y hearts upon contraction (particularly evident in KO2, right panel).

Video 2

Tβ4 knockout mice subjected to dobutamine stress testing, with high temporal cine MR imaging. The inotropic effects of dobutamine increase cardiac output via elevated heart rate and enhanced myocardial contractility. +/Y control mice (WT) decreased ESV with dobutamine treatment, as evidence of a normal contractile reserve. In contrast, -/Y (KO) mice already display small ESV at baseline, and this is only modestly reduced by dobutamine treatment. This test reveals that Tβ4 -/Y mice possess a limited contractile reserve.

Video 3

Tβ4 -/Y mice depend upon increasing heart rate and ejection fraction to maintain an effectively normal cardiac output. Bolus infusion of the β1-selective antagonist esmolol slowed heart rate and increased LV volumes in both +/Y (WT) and -/Y (KO) hearts. Whereas esmolol treatment markedly reduced SV in WT mice, KO mice were unaffected; cardiac output dropped significantly in KO mice.
